# Interactions Between *Streptococcus gordonii* and *Fusobacterium nucleatum* Altered Bacterial Transcriptional Profiling and Attenuated the Immune Responses of Macrophages

**DOI:** 10.3389/fcimb.2021.783323

**Published:** 2022-01-07

**Authors:** Tingjun Liu, Ruiqi Yang, Jiani Zhou, Xianjun Lu, Zijian Yuan, Xi Wei, Lihong Guo

**Affiliations:** Hospital of Stomatology, Guanghua School of Stomatology, Sun Yat-sen University, Guangdong Provincial Key Laboratory of Stomatology, Guangzhou, China

**Keywords:** dual RNA-seq, *F. nucleatum* subsp. *polymorphum*, interspecies coaggregation, macrophage killing, *S. gordonii*

## Abstract

Interspecies coaggregation promotes transcriptional changes in oral bacteria, affecting bacterial pathogenicity. *Streptococcus gordonii* (*S. gordonii*) and *Fusobacterium nucleatum* (*F. nucleatum*) are common oral inhabitants. The present study investigated the transcriptional profiling of *S. gordonii* and *F. nucleatum* subsp. *polymorphum* in response to the dual-species coaggregation using RNA-seq. Macrophages were infected with both species to explore the influence of bacterial coaggregation on both species’ abilities to survive within macrophages and induce inflammatory responses. Results indicated that, after the 30-min dual-species coaggregation, 116 genes were significantly up-regulated, and 151 genes were significantly down-regulated in *S. gordonii*; 97 genes were significantly down-regulated, and 114 genes were significantly up-regulated in *F. nucleatum* subsp. *polymorphum*. Multiple *S. gordonii* genes were involved in the biosynthesis and export of cell-wall proteins and carbohydrate metabolism. *F. nucleatum* subsp. *polymorphum* genes were mostly associated with translation and protein export. The coaggregation led to decreased expression levels of genes associated with lipopolysaccharide and peptidoglycan biosynthesis. Coaggregation between *S. gordonii* and *F. nucleatum* subsp. *polymorphum* significantly promoted both species’ intracellular survival within macrophages and attenuated the production of pro-inflammatory cytokines IL-6 and IL-1β. Physical interactions between these two species promoted a symbiotic lifestyle and repressed macrophage’s killing and pro-inflammatory responses.

## 1 Introduction

The oral microbiome comprises more than 700 bacterial species (Paster et al., 2006). Different bacterial species colonize close to each other in a symbiotic oral microbial community, establishing multi-species and highly structured biofilms (Stoodley et al., 2002). These functional biofilms often facilitate mutually beneficial metabolic activities among species, promoting greater good for multi-species homeostasis and survival (Møller et al., 1998). *Streptococcus gordonii* (*S. gordonii*) is one of the early colonizers of the oral cavity. It actively interacts with genetically different bacterial species and remains abundant in both supragingival and subgingival plaques in the healthy oral microbial community (Aas et al., 2005; Zeng et al., 2012). *Fusobacterium nucleatum* (*F. nucleatum*) is an anaerobic Gram-negative rod bacterium, serving as a critical bridge binding to early and late oral colonizers (Takemoto et al., 1995; Bolstad et al., 1996; Kaplan et al., 2009).

Microbial dysbiosis is one of the main driving factors that initiate periodontitis (Hajishengallis, 2015). An increased proportion of *F. nucleatum* was observed in diseased oral sites (gingivitis and periodontitis), and *F. nucleatum* was recognized as a putative periodontal pathogen (Socransky et al., 1998). *S. gordonii* used to be classified as an “accessory pathogen” because *S. gordonii* actively binds to keystone periodontal pathogens such as *Porphyromonas gingivalis*, and these interspecies interactions might promote the development of periodontal disease (Kuboniwa et al., 2017). Nevertheless, metagenomic sequencing analysis on subgingival samples revealed that *S. gordonii* was more abundant in subgingival plaque samples of healthy participants than in those of periodontitis patients, indicating that *S. gordonii* was associated with periodontal health (Socransky et al., 1998; Shi et al., 2020).

Coaggregation is a process of the cell-to-cell specific recognition between genetically distinct bacteria *via* specific molecules (Rickard et al., 2003). These specific molecules mainly consist of proteins (also known as adhesins) and polysaccharide moieties (often referred to as receptors) (Kolenbrander and Phucas, 1984). Coaggregation can also be mediated by adhesin–adhesin interactions or multiple interacting pairs of macromolecules (Katharios-Lanwermeyer et al., 2014). Extracellular polysaccharide (EPS) might participate in the coaggregation process, depending on the bacterial species involved (Katharios-Lanwermeyer et al., 2014). A previous study revealed that GtfB and GtfC of *S. mutans*, which contributed to EPS synthesis, promoted coaggregation of *S. mutans* with saliva isolate *Streptococcus agalactiae* (Liu et al., 2020). *S. gordonii* and *F. nucleatum* are a typical pair of coaggregated oral inhabitants. Several lines of evidence revealed strong coaggregation between *F. nucleatum* subspecies *nucleatum* and *S. gordonii* (Kaplan et al., 2009; Lima et al., 2017; Mutha et al., 2018). Previous research revealed that two fusobacterial molecules, including adhesin RadD and coaggregation mediating protein A, played an essential role in the coaggregation between *F. nucleatum* ATCC 23726 and *S. gordonii* V288 (Kaplan et al., 2009; Lima et al., 2017). This intergeneric physical contact induces signaling cascades and results in transcriptional changes of the attached species, affecting the bacterial virulence and survival in the oral cavity (Guo et al., 2014). Mutha et al. demonstrated that coaggregation between *S. gordonii* DL1 and *F. nucleatum* subspecies *nucleatum* induced significant transcriptional changes in both species (Mutha et al., 2018). *F. nucleatum* subspecies *polymorphum*, another pathogenic subspecies, has been frequently identified in periodontitis sites (Bolstad et al., 1996). However, the influence of *F. nucleatum* subsp. *polymorphum*-*S. gordonii* coaggregation on the transcriptional changes of both species needs further investigation, and investigations on this issue might further disclose the roles of coaggregation in microbial communications and pathogenicity.

Macrophages are active against invading pathogens in periodontitis lesions (Hajishengallis, 2015). In the *in vivo* oral microbial community, macrophages often interact with coaggregated bacterial species rather than single microbial species. Interspecies coaggregation, which promotes close intercellular communication and affects polymicrobial pathogenicity, has been shown to affect host innate immune responses (Li et al., 2015; Bor et al., 2016). Previous studies indicated that oral bacteria evolved multiple strategies to reduce sensitivity to macrophage killing, such as remodeling of membrane-bounded compartments and attenuation of bacterial virulence and immunogenicity (Bor et al., 2016; Mitchell et al., 2016; Croft et al., 2018; Xue et al., 2018). However, most of the past studies investigated the interactions of macrophages with *S. gordonii* and *F. nucleatum* separately (Cho et al., 2013; Kim et al., 2017; Chen et al., 2018; Croft et al., 2018; Liu et al., 2019; Wu et al., 2019), and few studies considered the effect of the interspecies coaggregation. The present study employed RNA-seq to study transcriptional changes of *S. gordonii* and *F. nucleatum* subsp. *polymorphum* in response to the dual-species coaggregation. The transcriptional profiling evaluated the influence of coaggregation on both species’ pathogenicity at the gene level. The study further investigated how bacterial coaggregation might affect both species’ abilities to survive within macrophages and trigger inflammatory responses, providing insight into roles of coaggregated *S. gordonii* and *F. nucleatum* subsp. *polymorphum* in inducing macrophages’ immune responses.

## 2 Materials and Methods

### 2.1 Bacterial Growth Conditions

*S. gordonii* DL1 was grown in Brain Heart Infusion (BHI) broth (Difco, USA). *F. nucleatum* subsp. *polymorphum* (ATCC10953) was grown in BHI broth supplemented with 0.5% yeast extract (Difco, USA), 1 μg/ml vitamin K (Sigma-Aldrich, USA), and 5 μg/ml hemin (Sigma-Aldrich, USA) (Ding and Tan, 2016). Both bacterial strains were grown under anaerobic conditions (85% N_2_, 10% H_2_, 5% CO_2_) at 37°C.

### 2.2 Coaggregation Assay

Intergeneric coaggregation was performed in coaggregation buffer (CAB) (150 mM NaCl, 1 mM Tris pH 8.0, 0.1 mM CaCl_2_, 0.1 mM MgCl_2_) as previously described (Cisar et al., 1979). *F. nucleatum* subsp. *polymorphum* and *S. gordonii* DL1 were grown until they reached late exponential growth phase. The optical density (OD_600nm_) of *F. nucleatum* subsp. *polymorphum* reached 0.80 (~10^9^ CFU), and the OD_600nm_ of *S. gordonii* DL1 was assessed to be 0.65 (~10^9^ CFU). Bacterial cells of each species were harvested, washed twice, and resuspended in CAB to a final concentration of ~2×10^9^ cells/ml. In the coaggregation group, equal numbers of cells from each species were pooled together in a new sterile reaction tube. The reaction tube was vortexed for 20 seconds to maximize bacterial physical contact and incubated anaerobically at 37°C for 10 to 30 min for coaggregation. The cells were then pelleted by centrifugation at 100×g for 1 min. The supernatant containing non-aggregated bacterial cells was carefully collected and measured for OD_600nm_ (OD_600nm(_ *_Sg_*_–_ *_Fnp_*_)_). In the autoaggregation groups, *F. nucleatum* subsp. *polymorphum* and *S. gordonii* cells were incubated separately in CAB to evaluate each species’ level of autoaggregation. The levels of interspecies binding at different time points were calculated as follows (Lima et al., 2017; Lima et al., 2019):


$$\text{Coaggregation level}=\;\frac{{\text{OD}}_{600\text{nm}\left(Sg\right)}+{\text{OD}}_{600\text{nm}\left(Fnp\right)}-{\text{OD}}_{600\text{nm}\left(Sg-Fnp\right)}}{{\text{OD}}_{600\text{nm}\left(Sg\right)}+{\text{OD}}_{600\text{nm}\left(Fnp\right)}}\times 100\%$$

OD_600nm(_ *_Sg_*_)_ and OD_600nm(_ *_Fnp_*_)_ represents the optical density of each individual species at 600nm, and OD_600nm(_ *_Sg-Fnp_*_)_ represents the optical density of the recovered supernatant after coaggregation. Pellets were frozen at -80°C and used for RNA extraction within 3 days. Three replicates were performed for this assay.

### 2.3 RNA Extraction

Total RNA of the coaggregated pellets and the monocultures of *F. nucleatum* subsp. *polymorphum* and *S. gordonii* DL1 was extracted using the hot phenol method and RNAprep Pure Tissue Kit according to the manufacturer’s instructions (Tiangen Biotech, Beijing, China). The extracted RNA was dissolved in 30-100 μl RNase-free water. RNA was quantified using NanoDrop-2000 (NanoDrop Technologies, DE, USA), and the integrity of RNA was determined using 2100 Bioanalyser (Agilent, CA, USA).

### 2.4 Library Preparation and Illumina Hiseq Sequencing

Library preparation and Illumina Hiseq sequencing were performed at Lianchuan Bio (Hangzhou, China). Five μg of total RNA from each sample was used. RNA-seq strand-specific libraries were prepared using the TruSeq™ RNA sample preparation kit (Illumina, CA, USA) according to the manufacturer’s instructions. Briefly, rRNA was removed using RiboZero rRNA removal kit (Illumina, CA, USA), and the remaining mRNA was fragmented using fragmentation buffer. After rRNA depletion, the first-strand cDNA was synthesized using random hexamer primers. Subsequently, the second-strand cDNA synthesis was performed. According to Illumina’s protocol, the fragments were then end repaired, added with poly(A) tails, and ligated to the Illumina-indexed adaptors. The second-strand cDNA was degraded using Uracil-DNA glycosylase (UNG). After purification, the product underwent PCR amplification using Phusion DNA polymerase (NEB) for 15 PCR cycles. The RNA-seq strand-specific library was then size-selected for cDNA target fragments of 200–300 bp on 2% low range ultra agarose. After quantified by TBS-380 Fluorometer (Turner BioSystems, CA, USA), the paired-end library was sequenced using Illumina NovaSeq 6000 platform (150bp×2).

### 2.5 Reads Quality Control and Reads Mapping to the Reference Genomes

The raw paired-end reads from Illumina sequencing were saved in the form of FASTQ files. Adapter sequences and low-quality reads (quality score < 20) were removed by Trimmomatic with parameters (SLIDINGWINDOW:4:15 MINLEN:75) (version 0.36 http://www.usadellab.org/cms/uploads/supplementary/Trimmomatic). Then clean reads were separately aligned to the reference genome with orientation mode using Rockhopper (http://cs.wellesley.edu/~btjaden/Rockhopper/) software. Gene expression levels were also analyzed by Rockhopper with default parameters. Due to the incomplete database annotations in the reference genome of *Fusobacterium nucleatum* subsp. *polymorphum* ATCC 10953, reads from *Fusobacterium nucleatum* subsp. *polymorphum* monoculture were mapped to *Fusobacterium nucleatum* subsp. *polymorphum* NCTC10562, the closest NCBI reference genome for better mapping and further analysis, and the results were denoted as “*Fnp*.” Reads from *S. gordonii* DL1 monoculture were mapped to *Streptococcus gordonii str. Challis substr.* CH1 reference genome (NCBI), and the results were denoted as “*Sg*.” Samples from the dual-species coaggregation group were mapped to *S. gordonii str. Challis substr.* CH1 reference genome (NCBI) in the first mapping round, and the results were designated as “CAB_*Sg*.” In the second round of mapping, samples from the dual-species coaggregating group were mapped to *Fusobacterium nucleatum* subsp. *polymorphum* NCTC10562 reference genome (NCBI), and the results were designated as “CAB_*Fnp*”.

### 2.6 Differential Expression Analysis and Functional Enrichment Analyses

To identify differentially expressed genes (DEGs) induced by dual-species coaggregation, the expression level for each transcript was quantified using the Transcripts Per Million mapped reads method. Comparisons between Group CAB_*Sg* and Group *Sg* were performed to explore coaggregation-induced DEGs of *S. gordonii* DL1. Group CAB_*Fnp* and Group *Fnp* were compared as well to investigate coaggregation-induced DEGs of *F. nucleatum* subsp. *polymorphum.* edgeR (https://bioconductor.org/packages/release/bioc/html/edgeR.html) was used for differential expression analysis. The DEGs were selected using the following criteria: the |log2(fold change)| was greater than 1, and the false discovery rate (FDR) should be less than 0.05.

To explore the functions of the DEGs, Gene Ontology (GO) functional enrichment analysis and Kyoto Encyclopedia of Genes and Genomes (KEGG) pathway enrichment analysis were carried out by Goatools (https://github.com/tanghaibao/Goatools) and KEGG Orthology-Based Annotation System (http://kobas.cbi.pku.edu.cn/home.do), respectively. DEGs were considered significantly enriched in GO terms and KEGG pathways if their Benjaminiand Hochberg-corrected *p* values were less than 0.05. The STRING version 11.0 database (https://string-db.org/) was used to identify functional interactions among DEGs.

### 2.7 Quantitative Reverse Transcription PCR (RT-qPCR)

RT-qPCR was used to validate the dual RNA-seq data. Total RNA of the coaggregated pellets and the monocultures was extracted and examined as previously described. RNA reverse transcription process was performed according to the manufacturer’s instructions (Hifair^®^ II 1st Strand cDNA Synthesis Kit, Yeasen Biotech, Shanghai, China). RT-qPCR was performed using Hieff^®^ qPCR SYBR Green Master Mix (Low Rox Plus) (Yeasen Biotech, Shanghai, China) following the manufacturer’s protocol. Relative expression was calculated by normalizing to the corresponding 16S rRNA gene transcripts. Three biological replicates were performed for all RT-qPCR reactions. PCR primers used in the present study are listed in Supplementary Materials (**Table S1**). Gene expression correlations between RT-qPCR results and RNA-seq results were evaluated by Pearson’s correlation coefficients using GraphPad Prism 8 (https://www.graphpad.com/scientific-software/prism/).

### 2.8 Short-Chain Fatty Acids (SCFAs) Production Detected by Gas Chromatography-Mass Spectrometry (GC-MS) Analysis

Supernatant samples of four different culture groups (monoculture, co-culture, and coaggregation) were collected. In the monoculture groups, bacterial cells of each species were harvested, washed twice, resuspended in CAB, and cultured anaerobically for 30 min. Bacterial cells were then pelleted by centrifugation at 12,000×g for 5 min. The supernatant was carefully discarded, and fresh growth media (BHI broth supplemented with 0.5% yeast extract, 1 μg/ml vitamin K, and 5 μg/ml hemin) was added to a final concentration of ~1×10^9^ cells/ml. In the co-culture group, equal numbers of both species were harvested, mixed together, washed twice, resuspended in PBS, and cultured anaerobically for 30 min. Bacterial cells were then pelleted and cultured in fresh growth media (~1×10^9^ cells/ml). In the coaggregation group, bacterial cells of each species were harvested and coaggregated as previously described. After 30-min coaggregation, pellets were collected by centrifugation at 100×g for 1 min, and the supernatant was carefully removed. Fresh growth media was added to a final concentration of ~1×10^9^ cells/ml. All groups were cultured anaerobically at 37°C for another 3 h. Supernatant samples were then collected by centrifugation at 12,000×g for 5 min and stored at -80°C and sent to APPLIED PROTEIN TECHNOLOGY (Shanghai, China) for GC-MS analysis which measured the concentrations of various types of SCFAs, including acetic acid, propanoic acid, isobutyric acid, butyric acid, hexanoic acid, and valeric acid.

### 2.9 Culture and Differentiation of THP-1 Cells

The human monocyte cell line THP-1 was kindly provided by Stem Cell Bank, Chinese Academy of Sciences (Shanghai, China). THP-1 monocytes were grown in RPMI 1640 supplemented with 10% fetal bovine serum (FBS) (Australian origin, Thermo Fisher Scientific, United States) in a CO_2_ incubator (5% CO_2_ humidified atmosphere at 37°C). Cells were treated with 50 ng/ml phorbol 12-myristate 13-acetate (PMA, Sigma-Aldrich, United States) for 48 h for macrophage differentiation. The THP-1 derived macrophages (dTHP-1 cells) were then grown in fresh RPMI 1640 supplemented with 10% FBS for 24 h in a CO_2_ incubator.

### 2.10 Co-Culture Experiments of dTHP-1 Cells and Bacterial Cells

Bacterial invasion assay and intercellular bacterial survival assay were performed as described previously (Croft et al., 2018; Xue et al., 2018). dTHP-1 cells were seeded in sterile Corning™ Costar™ flat bottom 6-well cell culture plates (1.0×10^6^ cell/well) and incubated for 12 h before use. dTHP-1 cells were infected with *S. gordonii* DL1 alone, *F. nucleatum* subsp. *polymorphum* alone, co-culture of the two species, and coaggregates of the two species, respectively (multiplicity of infection [MOI] was 10:1). We conducted a preliminary test to determine the CFUs of the coaggregates when the same volume of bacterial cells was used across all groups. In detail, the supernatant of the coaggregation group containing non-aggregating cells was removed. The pellets (coaggregates) were resuspended in PBS, and the tube was vigorously vortexed until no pellet was visible. A 100-μl aliquot of the solution was taken, subjected to serial dilutions in PBS (1:10), and seeded onto sheep blood agar plates as well as BHI agar plates. After the preliminary test, the volumes of bacterial cells used in the coaggregation group were adjusted to ensure that the final aggregates had similar numbers of bacterial cells with the mono-culture groups and met the MOI (10:1) requirement. After initial centrifugation (5 min at 250×g) to increase interactions between dTHP-1 cells and bacterial cells, the culture plates were incubated for 30 min in a CO_2_ incubator at 37°C. Cells were further divided into two groups: one is for bacterial invasion assay, and the other is for intercellular bacterial survival assay.

For bacterial invasion assay, dTHP-1 cells were washed five times extensively with sterile PBS. The supernatants were collected, spread onto 5% sheep blood agar plates, and incubated anaerobically for 3 days to validate that extracellular bacteria were wiped out. Cells were immediately lysed using 0.1% Triton X-100 (Beyotime, Shanghai, China). The sample of each well was serially diluted (1:10 dilution), spread onto 5% sheep blood agar plates and BHI agar plates, and incubated for 16-48 h to quantify the colony-forming units (CFUs) of “invading bacterial cells”.

For intercellular bacterial survival assay, dTHP-1 cells with invading bacteria were incubated for another 2 h before cell lysis. Briefly, dTHP-1 cells were washed twice with sterile PBS and cultured in fresh RPMI 1640 containing 10% FBS, gentamicin (300 μg/ml), and metronidazole (200 μg/ml) (Solarbio, Beijing, China) for 2 h to exterminate extracellular bacteria. dTHP-1 cells were then washed three times with sterile PBS. The supernatant of each group was collected and spread onto 5% sheep blood agar plates, and the plates were incubated anaerobically for 3 days to validate the killing of extracellular bacteria. dTHP-1 cells were lysed using 0.1% Triton X-100 (Beyotime, Shanghai, China). The sample of each well was vortexed for 10 seconds, serially diluted (1:10 dilution), spread onto 5% sheep blood agar plates and BHI agar plates, and incubated for 16-48 h to quantify the CFUs of “surviving bacterial cells”. All experiments were repeated at least three times.


$$\;\text{Survival rates }=\frac{{\text{CFUs}}_{\text{surviving bacterial cells}}}{{\text{CFUs}}_{\text{invading bacterial cells}}}\times 100\%$$

### 2.11 Cell Viability Assay

dTHP-1 cells were seeded in 96-well plates (1×10^4^ cells/well) and infected with 0.5 μl/ml lipopolysaccharide (LPS) (Solarbio, Beijing, China), PBS (negative control), the monoculture, the co-culture, and the coaggregates of *S. gordonii* and *F. nucleatum* subsp. *polymorphum*, respectively. After 2 h or 4 h, the plates were washed with PBS and cultured in fresh RPMI 1640 containing 10% FBS, gentamicin (300 μg/ml), and metronidazole (200 μg/ml) (Solarbio, Beijing, China) to kill extracellular bacteria. After 2 h, the plates were cultured in fresh RPMI 1640 containing 10% FBS, gentamicin (60 μg/ml), and metronidazole (40 μg/ml). Cell viability assay was performed using Cell Counting Kit-8 (CCK-8, Dojindo Laboratories, Kumamoto, Japan), and 10 μl CCK-8 was added into each well following the manufacturer’s instructions. The absorbance of the supernatant was recorded at 450nm using a microplate reader (Tecan, Reading, UK). dTHP-1 cells exposed to PBS served as negative control, and fresh RPMI 1640 containing 10% FBS, gentamicin (60 μg/ml), and metronidazole (40 μg/ml) without cells or bacteria served as blank controls. All groups were assessed in quintuplicate.


$$\text{Cell viability}=\;\frac{{\text{OD}}_{450\text{nm}\left(\text{experimental groups}\right)}-{\text{OD}}_{450\text{nm}\left(\text{blank control}\right)}}{{\text{OD}}_{450\text{nm}\left(\text{negative control}\right)}-{\text{OD}}_{450\text{nm}\left(\text{blank control}\right)}}\times 100\%$$

### 2.12 Enzyme-Linked Immunosorbent Assay (ELISA)

dTHP-1 cells were seeded in 6-well plates (1×10^6^ cells/well) and were infected with 0.5 μl/ml LPS (Solarbio, Beijing, China), PBS, the monoculture, the co-culture, and the coaggregates of *S. gordonii* and *F. nucleatum* subsp. *polymorphum*, respectively. After 2 h or 4 h, the plates were washed with PBS and cultured in fresh RPMI 1640 containing 10% FBS, gentamicin (300 μg/ml), and metronidazole (200 μg/ml) (Solarbio, Beijing, China) to kill extracellular bacteria. After 2 h, the plates were cultured in fresh RPMI 1640 containing 10% FBS, gentamicin (60 μg/ml), and metronidazole (40 μg/ml). Supernatants of samples collected at different time points were collected by centrifugation at 3,000 rpm (20 min, 4°C). Pro-inflammatory cytokines IL-1β, IL-6, and TNF-α were analyzed using ELISA kits (Neobioscience, Shenzhen, China) according to the manufacturer’s instructions.

### 2.13 Statistical Analysis

Regarding results other than the transcriptional analysis, statistically significant differences (*p* < 0.05) were determined by One-Way analysis of variance with *post-hoc* Dunnett’s test.

## 3 Results

### 3.1 *F. nucleatum* subsp. *polymorphum* Coaggregated Strongly With *S. gordonii*

**Figure 1:**
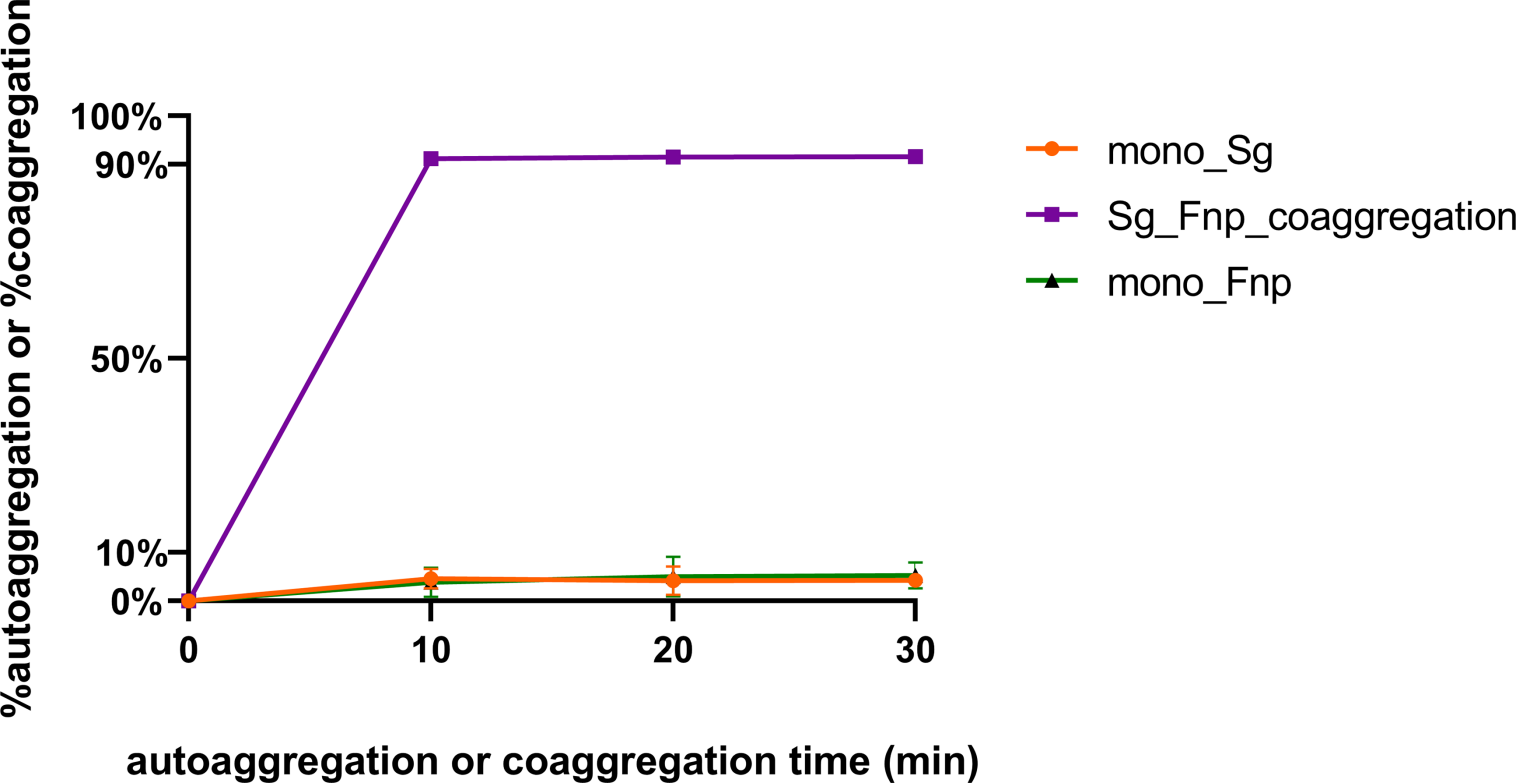
*S. gordonii* has a strong coaggregation ability with *F. nucleatum*. *S. gordonii* (Sg) and *F. nucleatum* subsp. *polymorphum* (Fnp) coaggregated in CAB for 10 to 30 min (Group Sg_Fnp_coaggregation). In addition, each species was resuspended in CAB and incubated individually to assess the levels of autoaggregation (Group mono_Sg and Group mono_Fnp). Results were shown as the coaggregation or autoaggregation rate of each group. Data at each time point represent the mean ± standard deviation (S.D.) of the results of three independent assays.

Coaggregation of *F. nucleatum* with other bacterial species is a highly specific process, and its subspecies exhibited varied coaggregation abilities (Hojo et al., 2009; Guo et al., 2017). The level of physical adherence between *F. nucleatum* subsp. *polymorphum* and *S. gordonii* was assessed in the study. Results of the coaggregation assay showed that the interspecies binding between *F. nucleatum* subsp. *polymorphum* and *S. gordonii* cells in CAB occurred quickly within 10 min and remained stable for the next 20 min (**Figure 1**). In addition, the level of dual-species coaggregation was high (~91%), while the level of autoaggregation of each species was markedly low (~10%). The present results indicated that *F. nucleatum* subsp. *polymorphum* and *S. gordonii* cells could extensively adhere to each other.

### 3.2 Read Pre-Processing and Alignment

In total, 57.35 million raw reads and 38.75 million clean reads were produced by the three coaggregated *F. nucleatum* subsp. *polymorphum* and *S. gordonii* biological replicates. Among these coaggregated biological replicates, an average of 32.54% clean reads were mapped to the *F. nucleatum* subsp. *polymorphum* reference genome, and 58.47% clean reads were mapped to the *S. gordonii* reference genome. In monoculture samples, three biological replicates of *F. nucleatum* subsp. *polymorphum* produced 47.26 million raw reads and 31.69 million clean reads, and an average of 88.12% clean reads were mapped to reference genomes of *F. nucleatum* subsp. *polymorphum*. Three biological replicates of *S. gordonii* yielded 45.0 million raw reads and 32.18 million clean reads, and an average of 92.76% clean reads were mapped to reference genomes of *S. gordonii*.

**Figure 2:**
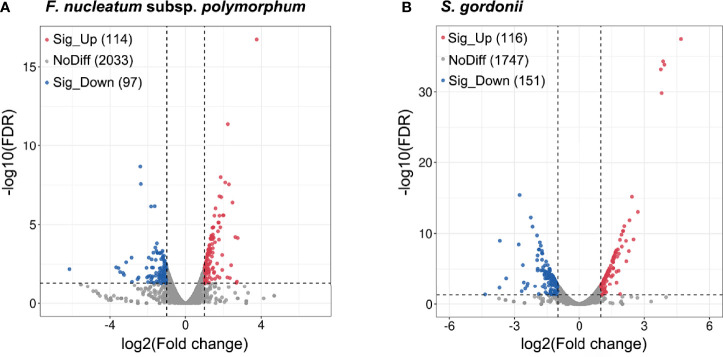
DEGs of *F. nucleatum* subsp. *polymorphum* and *S. gorondii* DL1 in response to the dual-species coaggregation. The x-axis shows the log2(Fold change) values, and the y-axis displays the negative logarithm of FDR to base 10. DEGs with fold changes ≥ 2 in coaggregate cultures compared with monocultures were considered “up-regulated”, and those with fold changes ≤ -2 were considered “down-regulated”. The blue dots represent significantly down-regulated genes, and the red dots correspond to significantly up-regulated genes (*p* < 0.05).

Analysis of differential gene expression revealed that a total of 267 *S. gordonii* genes and 211 F*. nucleatum* subsp. *polymorphum* genes were differentially expressed (*p* < 0.05). Volcano plots of DEGs in the two different comparisons illustrated distinct transcriptional profiles (**Figure 2**).

### 3.3 RT-qPCR Validation of RNA-Seq Data

**Figure 3:**
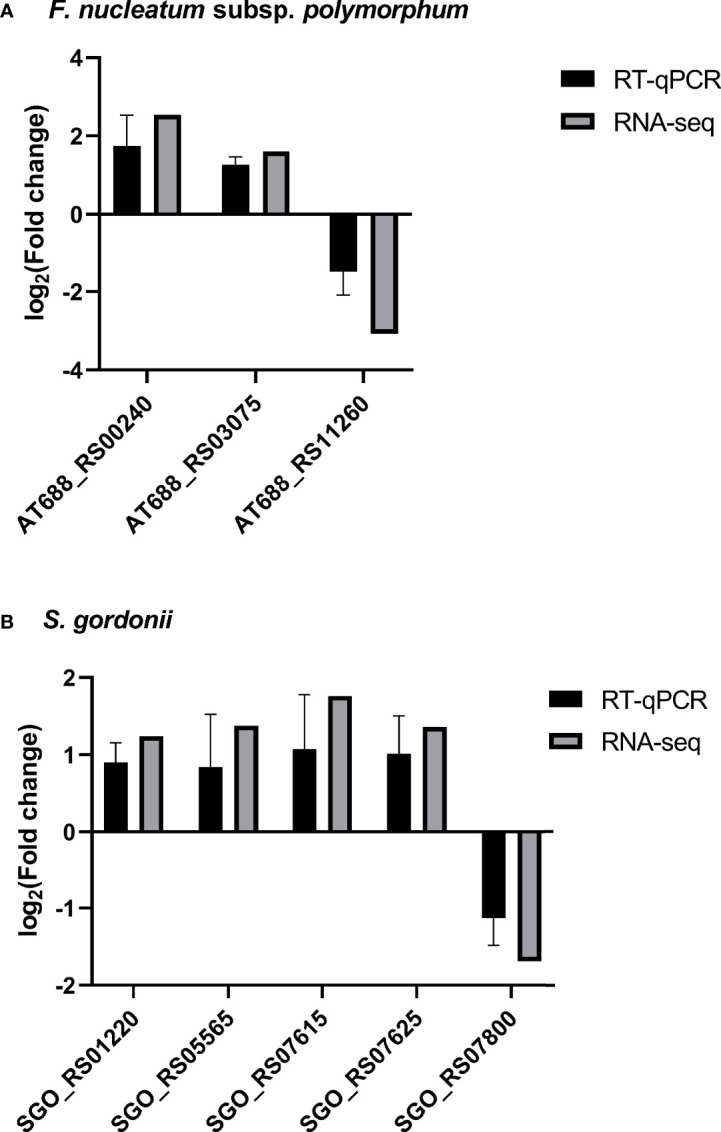
RT-qPCR validation of RNA-seq results. The expression of four *F. nucleatum* subsp. *polymorphum* DEGs and five *S. gordonii* DEGs were assessed by RT-qPCR, and the results were normalized to the corresponding 16S rRNA expression. Data represent the mean ± S.D. of the results of three independent assays. It is noticeable that there are strong correlations between expression levels of each gene using two different techniques.

A group of DEGs of each species was examined by RT-qPCR (**Figure 3**). Gene expression levels were normalized to 16S rRNA expression of the corresponding species. The patterns of gene expression by dual RNA-seq and RT-qPCR were similar. There was a strong correlation between the two data sets in each species (Pearson’s correlation coefficients were 0.9993 for *S. gordonii* DEGs and 0.9952 for *F. nucleatum* subsp. *polymorphum* DEGs, *p*<0.02).

### 3.4 Transcriptome Analysis of *S. gordonii* DL1 in Response to Coaggregation With *F. nucleatum* subsp. *polymorphum*

#### 3.4.1 DEGs of *S. gordonii*

In total, 267 genes of *S. gordonii* had significantly altered expression levels in response to coaggregation: 116 genes were up-regulated, and 151 genes were down-regulated. **Table S2** shows *S. gordonii* DEGs with key functions described in the GO knowledgebase. Key functions of the up-regulated DEGs included carbohydrate (derivative) metabolism (15 genes), phosphotransferase system (PTS) (8 genes), transcription (7 genes), membrane/cell wall-associated proteins (5 genes), pyruvate metabolism (4 genes), protein metabolism (4 genes), and ATP-binding cassette (ABC) transporter (3 genes). Key functions of the down-regulated DEGs included transcription (16 genes), ABC transporter (11 genes), fatty acid biosynthesis (5 genes), and arginine biosynthesis and metabolism (4 genes).

#### 3.4.2 Functional Enrichment Analysis

To further explore the functional characteristics of DEGs, we performed GO enrichment analysis using Goatools (https://github.com/tanghaibao/GOatools). In total, DEGs were enriched in 28 GO terms (*p* ≤ 0.05). Noticeably, 10 DEGs were highly clustered in 6 interrelated biological processes: carbohydrate biosynthetic process (GO:0016051), cellular polysaccharide metabolic process (GO:0044264), polysaccharide metabolic process (GO:0005976), polysaccharide biosynthetic process (GO:0000271), cellular polysaccharide biosynthetic process (GO:0033692), and cellular carbohydrate biosynthetic process (GO:0034637). Those 10 associated DEGs are listed in **Table S3**: only one gene was down-regulated, and the gene is related to lipid metabolism; all the other DEGs were up-regulated, and they were mostly involved in carbohydrate biosynthesis and metabolism.

To better elucidate key functional pathways in which DEGs were involved, we performed KEGG pathway enrichment analysis. DEGs were enriched in multiple pathways, including the pathways of PTS (9 DEGs), starch and sucrose metabolism (7 DEGs), fatty acid biosynthesis (5 DEGs), and pyruvate metabolism (4 DEGs) (**Table S4**).

#### 3.4.3 Protein-Protein Interaction (PPI) Network Analysis

**Figure 4:**
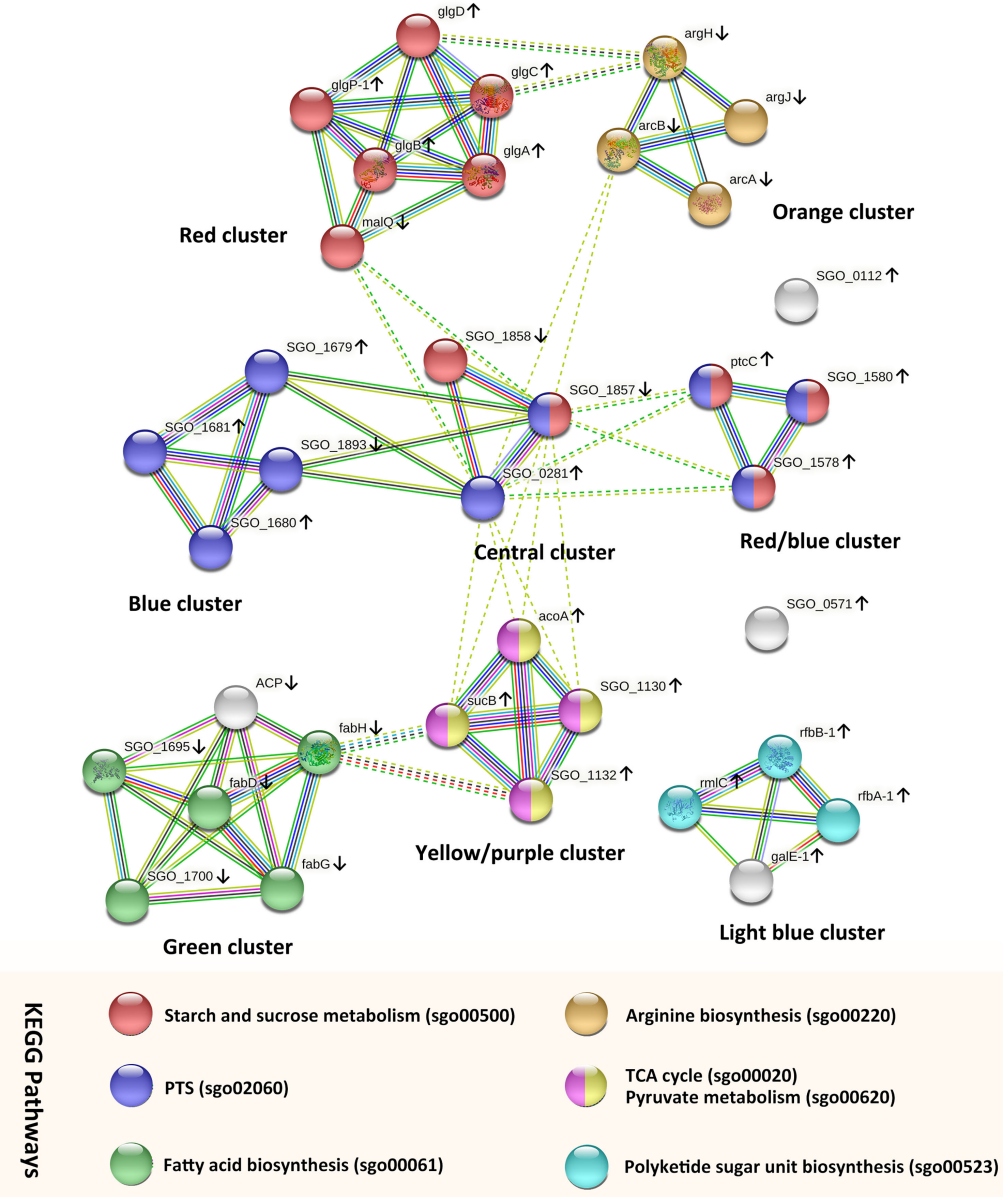
PPI networks analysis of *S. gordonii* DEGs that were enriched in the KEGG pathways or GO terms. Each node represents a gene. The up arrows and down arrows beside the gene symbols indicate the upregulation and downregulation of the gene, respectively. Nodes were clustered into eight groups, and the color of each colored node stands for a KEGG pathway as illustrated in the figure. Interactions between genes are displayed by colored edges (lines), and each color corresponds to a specific interaction source of evidence, such as curated databases (light blue), experimentally determined (purple), textmining (yellow-green), neighborhood (green), gene fusions (red), co‐occurrence (blue), and coexpression (black).

To further analyze and predict the interactions of DEGs enriched in key GO functions or KEGG pathways, we performed PPI networks functional enrichment analysis of DEGs. We input genes listed in **Tables S3** and **S4** into the STRING database. The results, visualized in a network graphic, showed that 34 out of 36 input genes were grouped into 8 clusters (**Figure 4**). Analysis results indicated that the network had significantly more interactions than expected, and the associated proteins were biologically connected (*p* < 0.001).

The genes in the central cluster include SGO_0281 (SGO_RS01385, PTS glucose transporter subunit IIA, up-regulated), SGO_1857 (SGO_RS09095, PTS beta-glucoside transporter subunit IIBCA, down-regulated), and SGO_1858 (SGO_RS09100, sucrose-6-phosphate hydrolase, down-regulated). These genes encode proteins that participate in the carbohydrate transport process, and they potentially interacted with genes in the other 5 clusters. In addition, genes in the red cluster were all involved in the KEGG pathway of starch and sucrose metabolism (sgo00500). All but one of the genes were up-regulated with fold changes between 2.53 and 3.18, and *malQ* (SGO_RS00525, 4-alpha-glucanotransferase) was the only down-regulated gene. Genes in the blue cluster were all mapped to the PTS pathway (sgo02060) and up-regulated. Genes in the red/blue cluster were all up-regulated, and they were enriched in the pathways of starch and sucrose metabolism and PTS. In each of those clusters, the analysis identified strong evidence of connections between genes.

Genes in the yellow/purple cluster were enriched in the pathways of the citrate cycle (TCA cycle) (sgo00020) and pyruvate metabolism (sgo00620). All genes were up-regulated, including SGO_1130 (SGO_RS05555, dihydrolipoyl dehydrogenase), *acoA* (SGO_RS05570, thiamine pyrophosphate-dependent dehydrogenase E1 component subunit alpha), *sucB* (SGO_RS05560, dihydrolipoamide acetyltransferase), and SGO_1132 (SGO_RS05565, alpha-ketoacid dehydrogenase subunit beta). Genes *sucB* and SGO_1132 potentially interacted with *fabH* (SGO_RS08325, ketoacyl-ACP synthase III) from the green cluster.

All but one of the genes in the green cluster were enriched in the pathway of fatty acid biosynthesis (sgo00061). The gene *acp* (SGO_RS08320, acyl carrier protein) encodes a protein that serves as a growing fatty acid chain carrier. Although the STRING database analysis failed to recognize the involvement of gene *acp* in the pathway of fatty acid biosynthesis, the search result of *acp* in the UniProt database (https://www.uniprot.org/uniprot/A8AYW4) indicated that *acp* also participated in the pathway of fatty acid biosynthesis. All genes were down-regulated with fold changes ranged from -2.17 to -3.08. In addition, DEGs in the orange cluster were all involved in the arginine biosynthetic process and were down-regulated with fold changes ranged from -2.27 to -3.45 (SGO_RS07800, *argF*; SGO_RS00870, *argH*; SGO_RS07680, *argJ*; and SGO_RS07805, *arcA*).

Three genes in the light blue cluster were involved in the pathway of polyketide sugar unit biosynthesis (sgo00523): *rfbB-1* (SGO_RS04960, dTDP-glucose 4,6-dehydratase), *rfbA-1* (SGO_RS04950, glucose-1-phosphate thymidylyltransferase RfbA), and *rmlC* (SGO_RS04955, dTDP-4-dehydrorhamnose 3,5-epimerase family protein). The gene *galE-1* (SGO_RS04965, UDP-glucose 4-epimerase GalE) was involved in the pathway of galactose metabolism. All genes were up-regulated with fold changes between 2.54 and 3.14.

### 3.5 Transcriptome Analysis of *F. nucleatum* subsp. *polymorphum* Following Coaggregation

#### 3.5.1. DEGs of *F. nucleatum* subsp. *polymorphum*

In total, 211 genes in *F. nucleatum* subsp. *polymorphum* had significantly altered expression levels in response to coaggregation with *S. gordonii*: 114 genes were up-regulated, and 97 genes were down-regulated. **Table S5** shows *F. nucleatum* subsp. *polymorphum* DEGs with key functions described in the GO knowledgebase. Key functions of DEGs included translation (18 genes), nucleic acid metabolism (6 genes), ABC transporter (5 genes), protein export (4 genes), oxidoreductase activity (3 genes), and DNA repair (2 genes).

#### 3.5.2 Functional Enrichment Analysis

GO enrichment analysis demonstrated that DEGs were enriched in 42 biological processes, 12 molecular functions, and 19 cellular components (*p* ≤ 0.05). A total of 14 DEGs were highly clustered in 5 interrelated biological processes: ribosome assembly (GO:0042255), organelle assembly (GO:0070925), cellular protein-containing complex assembly (GO:0034622), ribonucleoprotein complex assembly (GO:0022618), and ribonucleoprotein complex subunit organization (GO:0071826). The associated DEGs are listed in **Table S6**. All but one of the DEGs were up-regulated. Results of KEGG enrichment analysis revealed two significantly enriched pathways (*p* ≤ 0.05): translation (18 DEGs) and protein export (4 DEGs). All but one of the DEGs were up-regulated and are listed in **Table S7**.

#### 3.5.3 PPI Network Analysis

**Figure 5:**
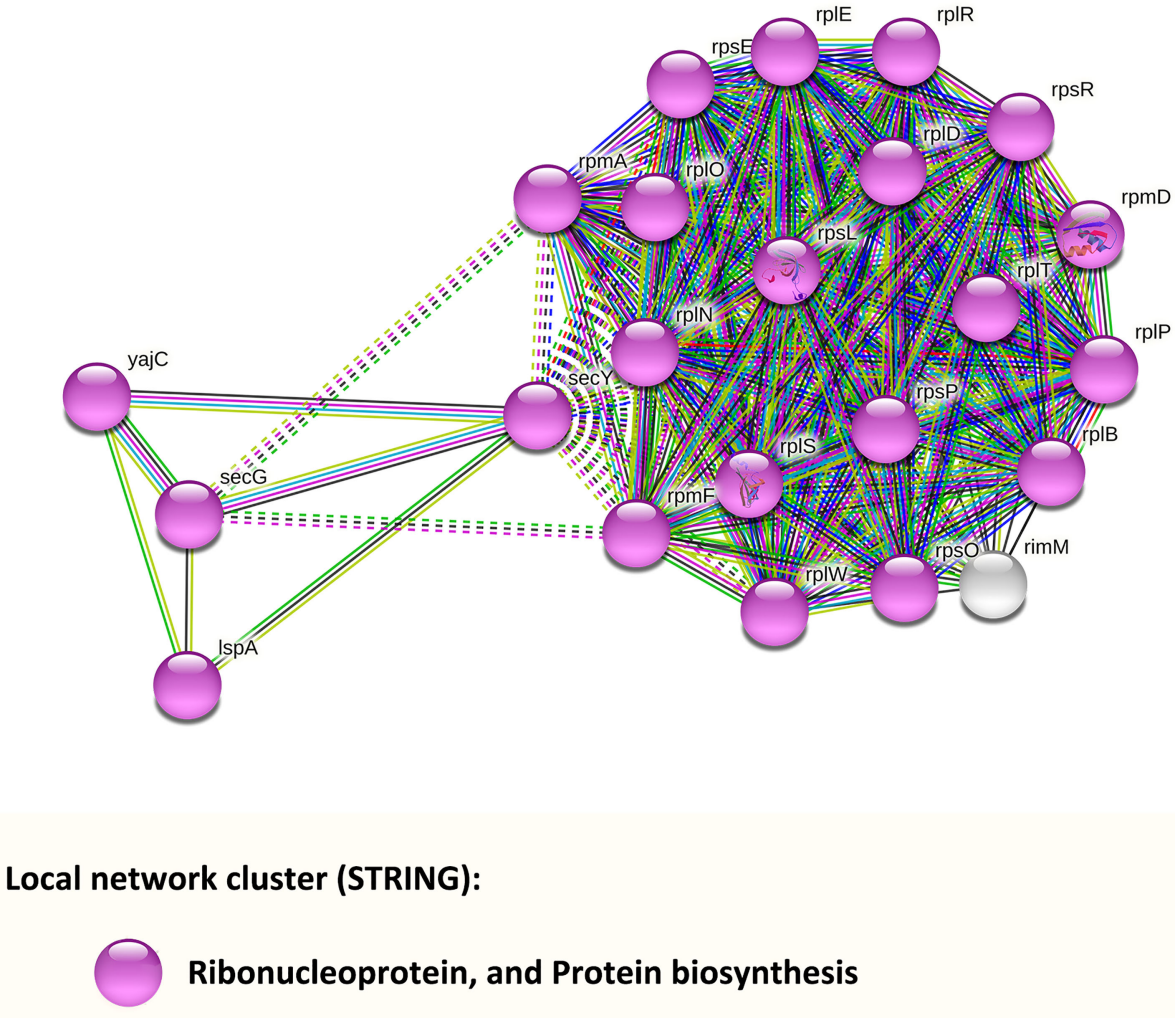
PPI networks analysis of *F. nucleatum* subsp. *polymorphum* DEGs that were enriched in the KEGG pathways or GO terms. Each node represents a gene, and all genes were down-regulated. The color of each colored node stands for a keyword of the gene identified in the UniProt database.

To further explore PPI, we input genes from **Tables S6** and **S7** into the STRING database (**Figure 5**). Results indicated that the network had significantly more interactions than expected (*p* < 0.001). Twenty-two of the input genes, displayed in purple, belong to the local network cluster of the ribonucleoprotein and protein biosynthesis. Among them, four genes displayed in the left part of **Figure 5** were involved in protein export: *secY* (AT688_RS02140, preprotein translocase subunit SecY, up-regulated), *yajC* (AT688_RS04215, preprotein translocase subunit YajC, up-regulated), *secG* (AT688_RS08695, preprotein translocase subunit SecG, up-regulated), and *lspA* (AT688_RS11260, lipoprotein signal peptidase, down-regulated).

### 3.6 Coaggregated *S. gordonii* and *F. nucleatum* subsp. *polymorphum* Secreted Significantly Low Amount of Propanoic Acid and Butyric Acid

**Figure 6:**
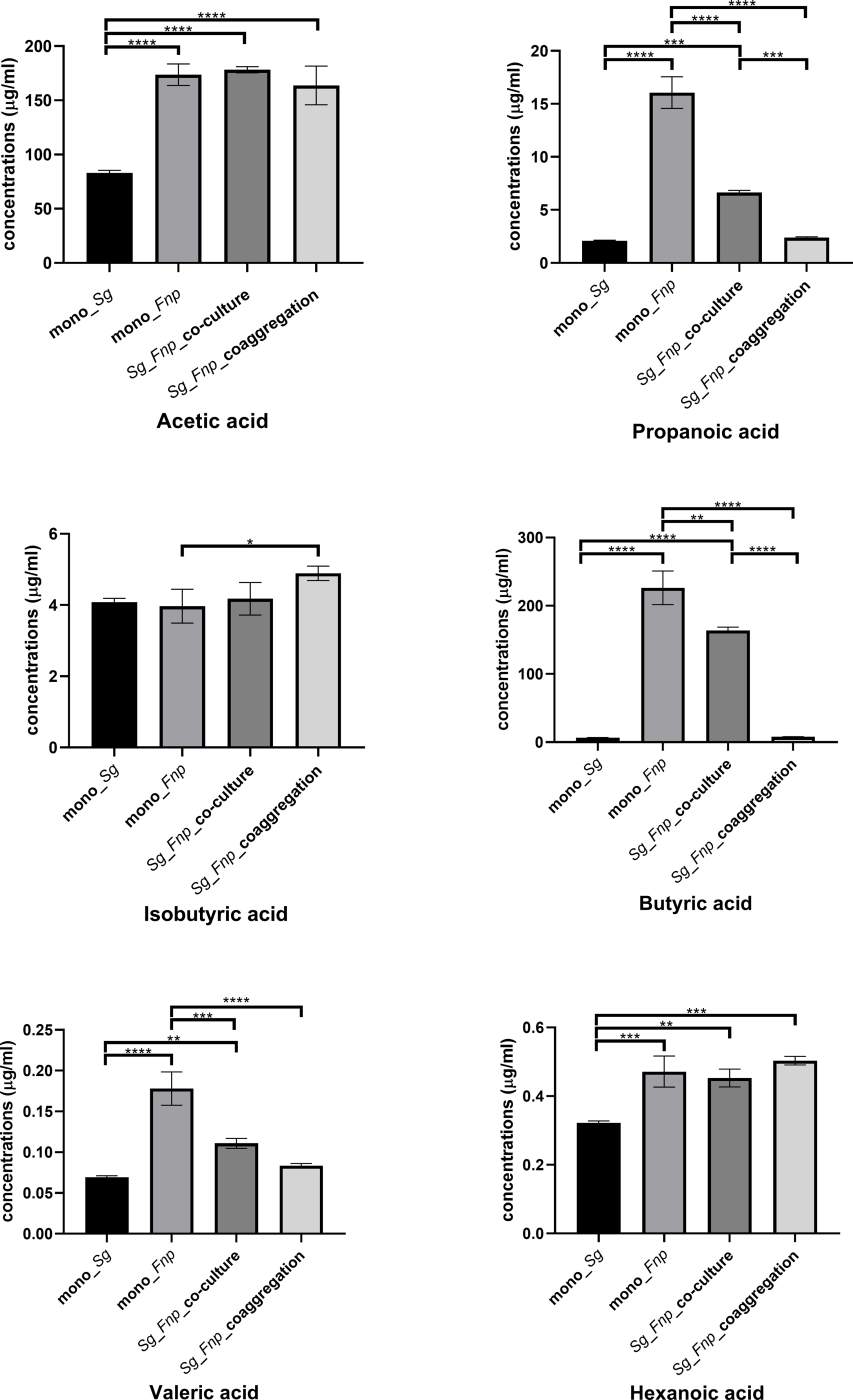
Concentrations of various SCFAs in four groups. Data represent the mean ± S.D. of the results of three independent assays. The asterisks indicate significant difference between groups (*p* < 0.05).

*S. gordonii* and *F. nucleatum* subsp. *polymorphum* do not automatically coaggregate in the co-culture group which serves as control. We previously treated *S. gordonii* and *F. nucleatum* subsp. *polymorphum* with CAB buffer lacking calcium, and these two strains still coaggregated extensively. The coaggregation levels were similar to those occurring in the original CAB buffer. Coaggregation between *S. gordonii* and *F. nucleatum* is likely mediated *via* multiple pairs of macromolecules, including adhesins and protein-receptors (Kaplan et al., 2009; Lima et al., 2017). Calcium might play a less important role in the coaggregation between these two species. PBS is a buffer solution commonly used in biological research, and *S. gordonii* and *F. nucleatum* subsp. *polymorphum* did not automatically coaggregate in PBS. Therefore, PBS, instead of CAB without calcium, was used as a buffer in the control group. Results showed that, compared with the mono-species groups and dual-species co-culture group, the dual-species coaggregation group exhibited significantly decreased levels of propanoic acid and butyric acid (**Figure 6**). Results indicated that dual-species coaggregation greatly curtailed the overall production of propanoic acid and butyric acid.

### 3.7 Coaggregation Between *F. nucleatum* subsp. *polymorphum* and *S. gordonii* Enhanced Both Species’ Survivability Within dTHP-1 Cells

**Figure 7:**
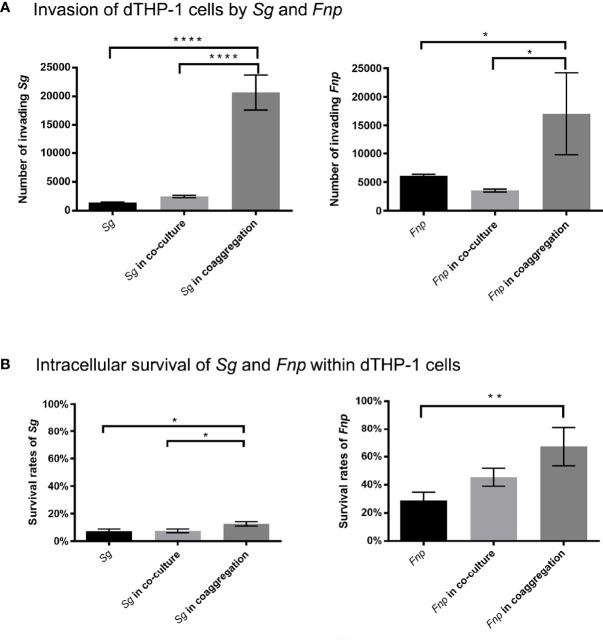
Co-culture experiments of dTHP-1 cells and bacterial cells. **(A)** shows the results of invasion of dTHP-1 cells by *F. nucleatum* subsp. *polymorphum* (*Fnp*) and *S. gordonii* (*Sg*). The monocultures, dual-species co-culture, and dual-species coaggregates of *F. nucleatum* subsp. *polymorphum* (*Fnp*) and *S. gordonii* (*Sg*) infected dTHP-1 cells, respectively, at an MOI of 10:1 for 30 min. Bacterial cells that invaded dTHP-1 cells were recovered. **(B)** shows the intracellular survival rates of *Fnp* and *Sg* within dTHP-1 cells. After 30 min incubation of bacteria and dTHP-1 cells, extracellular bacteria were exterminated by extensive wash and use of gentamicin, and dTHP-1 cells with invading bacteria were incubated for another two hours before cell lysis. The survival rates were determined by dividing the number of surviving bacterial cells by the number of invading bacterial cells. Data represent the mean ± S.D. of the results of three independent assays. The asterisks indicate significant difference between groups (*p* < 0.05).

To investigate the effect of dual-species coaggregation on both species’ invading and intracellular survival ability, we treated dTHP-1 cells with live *S. gordonii* cells, live *F. nucleatum* subsp. *polymorphum* cells, co-culture of the two species, and coaggregates of the two species, respectively. The bacterial cells that invaded and survived within dTHP-1 cells were counted, respectively. *F. nucleatum* subsp. *polymorphum* formed pinpoint circular colonies on blood agar plates, while *S. gordonii* formed grayish-white, larger, and smooth colonies on blood agar plates. In addition, we reconfirmed the CFUs of *S. gordonii* by counting the number of bacterial colonies on BHI agar plates because only *S. gordonii* could grow on the surfaces of BHI agar plates. Results revealed that significantly more *F. nucleatum* subsp. *polymorphum* cells and *S. gordonii* cells invaded macrophages in the coaggregation group compared with the co-culture group and the mono-species groups (**Figure 7A**). In addition, the survival rates of both species were significantly higher in the coaggregation group compared with other groups (**Figure 7B**).

### 3.8 Coaggregation Between *F. nucleatum* subsp. *polymorphum* and *S. gordonii* Markedly Reduced Pro-Inflammatory Responses of dTHP-1 Cells

**Figure 8:**
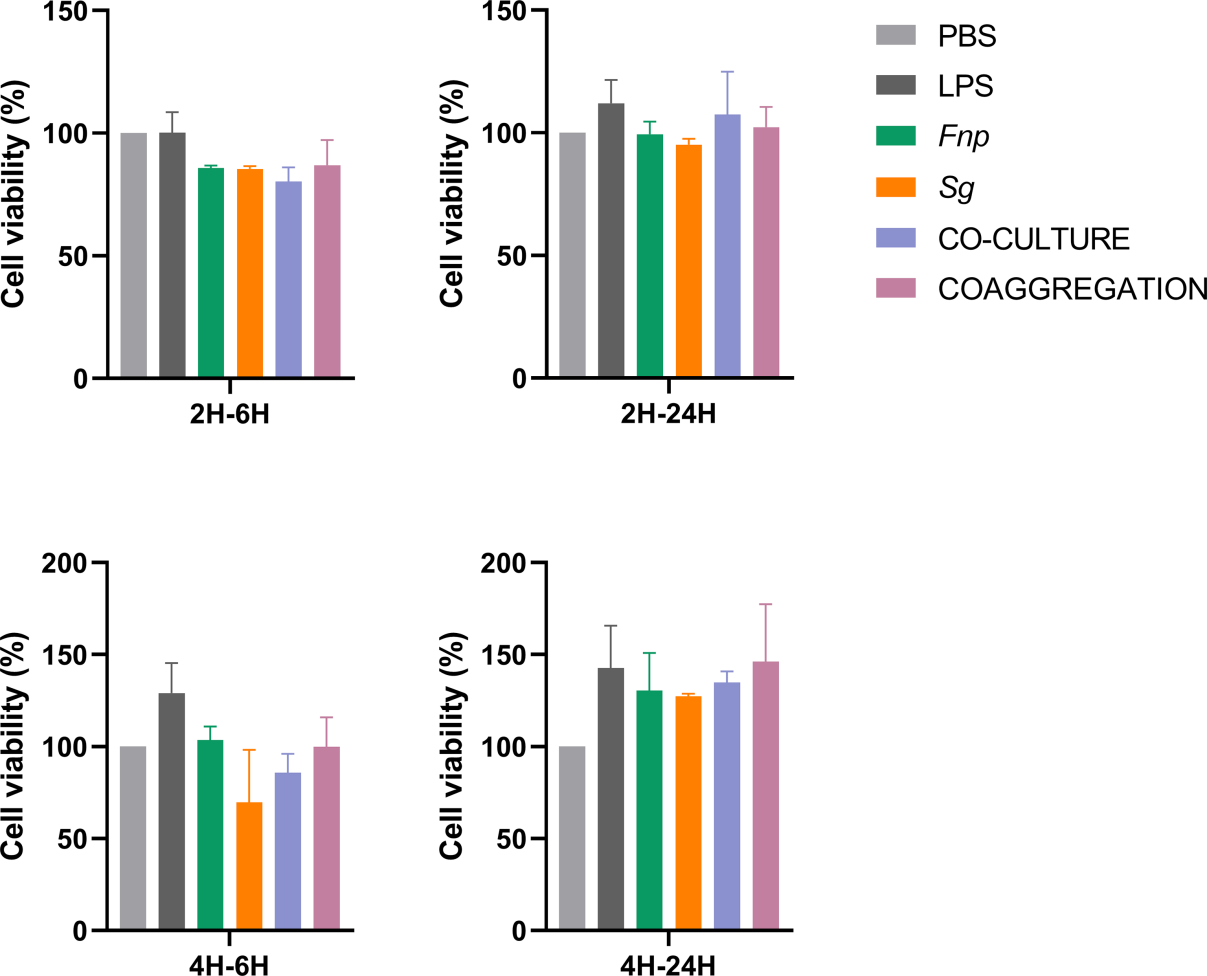
Cell viability of dTHP-1 cells following bacterial infection. Data represent the mean ± S.D. of the results of three independent assays.

**Figure 9:**
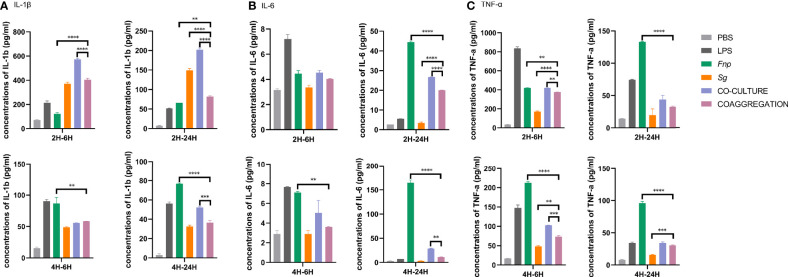
Secretion of pro-inflammatory cytokines by dTHP-1 cells. Data represent the mean ± S.D. of the results of three independent assays. The asterisks indicate significant difference between groups (*p* < 0.05).

Different time points were chosen according to relevant published studies (Bor et al., 2016; Xu et al., 2018; Periselneris et al., 2019). After 2 h or 4 h incubation time, extracellular bacteria were wiped out, and macrophages were incubated for another 6 h or 24 h. 6 h represents the early stage of cytokine production, and 24 h refers to the late stage of cytokine production. The time points chosen in the host cell viability assay was matched with those used in the cytokine production assay. Results of CCK-8 assay revealed that dTHP-1 cells infected with the coaggregates of *F. nucleatum* subsp. *polymorphum* and *S. gordonii* had similar cell viabilities with those infected with monocultures or the co-culture of both species at both 6 h and 24 h post infection (**Figure 8**). ELISA results demonstrated that dTHP-1 cells infected with the coaggregates of *F. nucleatum* subsp. *polymorphum* and *S. gordonii* secreted significantly lower levels of IL-1β and IL-6 at 24 h post infection compared with those infected with the co-culture of both species. The levels of TNF-α were similar between the co-culture group and the co-aggregation group at 24 h post infection (**Figure 9**).

## 4 Discussion

Streptococci are the primary initial bacterial inhabitants in the oral cavity. They actively interact with a wide array of salivary components and microorganisms and facilitate the colonization of subsequent species (Rogers et al., 2001; Abranches et al., 2018). Interspecies coaggregation promotes close spatial vicinity, allowing intergeneric signaling in micro distances and inducing genetic and phenotypic changes of partner species (Kolenbrander, 2000). Mutha et al. coaggregated *S. gordonii* DL1 and *F. nucleatum* subsp. *nucleatum* in human saliva for 30 minutes and analyzed genes that might play important roles in developing dual-species communities (Mutha et al., 2018). They found that only 16 genes were regulated following coaggregation in *F. nucleatum* subsp. *nucleatum* genes. Most of the genes were involved in sialic acid uptake and catabolism, and others were associated with transport of amino acid or lipid, catalytic activity or phosphorous metabolic process, and unknown functions. A gene cluster of *S. gordonii* associated with PTS-mediated uptake of lactose and galactose was significantly down-regulated following coaggregation.

The present study investigated interactions of another *F. nucleatum* subspecies with *S. gordonii*. *F. nucleatum* subsp. *polymorphum* has been frequently detected in periodontitis sites (Bolstad et al., 1996; Gmür et al., 2006). This subspecies exhibited high levels of interspecies coaggregation with *S. gordonii*. Dual RNA-seq unveiled subspecies-specific responses of *F. nucleatum* towards coaggregation with *S. gordonii*. Multiple DEGs of *F. nucleatum* subsp. *polymorphum* were up-regulated, and they were associated with nucleic acid metabolism, translation, and protein export. *S. gordonii* actively up-regulated genes associated with cell binding, indicating that *S. gordonii* enhanced its adherence ability during physical interplay with *F. nucleatum* subsp. *polymorphum*. Furthermore, DEGs of *S. gordonii* were enriched in the pathways of carbohydrate transport and metabolism as well as arginine biosynthesis and metabolism. Interestingly, the study discovered that genes involved in bacterial virulence and immunogenicity were affected, including *S. gordonii* DEGs associated with the pyruvate metabolism and peptidoglycan biosynthesis as well as *F. nucleatum* subsp. *polymorphum* DEGs involved in the biosynthesis of LPS and peptidoglycan. Moreover, the transcriptome results revealed that *S. gordonii* DEGs involved in the pathway of fatty acid biosynthesis were down-regulated. GC-MS analysis of SCFAs revealed that the coaggregates of *S. gordonii* and *F. nucleatum* subsp. *polymorphum* produced markedly lower levels of propanoic acid and butyric acid compared with the co-culture of both species.

Hence, we further explored the interactions of both species with macrophages. Results demonstrated that, in the dual-species coaggregation group, both species showed improved intracellular survival abilities and attenuated macrophages’ pro-inflammatory responses. The present study indicated that coaggregation between *S. gordonii* and *F. nucleatum* subsp. *polymorphum* contributed to both species’ symbiotic survival within macrophages and mitigated macrophages’ pro-inflammatory responses. One of the mechanisms might be the downregulation of multiple DEGs associated with bacterial immunogenicity and upregulation of DEGs related to oxidative stress resistance as indicated in the transcriptome results; another plausible regulation is through the reduced production of propanoic acid and butyric acid by coaggregated species.

### 4.1 Analysis of the Transcriptional Profiling of *S. gordonii*

#### 4.1.1 Most DEGs That Encode Membrane- and Cell Wall-Associated Proteins Were Up-Regulated

The transcriptome results indicated that dual-species coaggregation up-regulated DEGs associated with protein export systems and might enhance adherence of *S. gordonii* to *F. nucleatum* subsp. *polymorphum*. Bacteria export proteins from the biosynthesis sites, across the cell membranes, to the outer side of the cell wall. Proteins that anchored to the cell surface provide ideal sites for interspecies interactions (Rigel and Braunstein, 2008). In general, Gram-positive bacteria have two conventional protein export systems, namely the general secretion pathway and the twin-arginine translocation pathway. Besides, *S. gordonii* possesses an accessory secretion system (the secA2 system) that contributes to the transport of extracellular proteins (Rigel and Braunstein, 2008). The SecA2 system consists of integral membrane protein SecY2, membrane protein SecA2, and accessory secretion proteins 1 to 5 (Asp1 to Asp5) (Spencer et al., 2019). Results revealed that the gene (SGO_RS04755) responsible for encoding SecY2 was up-regulated with a fold change of 1.63 following coaggregation. Genes that encode all Asps but Asp4 (SGO_RS04760, SGO_RS04765, SGO_RS04770, and SGO_RS04795) were up-regulated with fold changes ranged from 1.36 to 2.67. Previous research indicated that the secA2 system of *S. gordonii* was used to export large serine-rich glycoproteins that adhered to the cell surface and promoted bacterial adhering ability (Bensing and Sullam, 2002; Rigel and Braunstein, 2008).

In addition to the genes associated with the protein export system, we identified five up-regulated DEGs that encode membrane- or cell wall-associated proteins (**Table S2**). Two of them (SGO_RS09805 and SGO_RS00535) encode Leu-Pro-x-Thr-Gly (LPxTG; x denotes any amino acid) cell wall anchor domain-containing proteins. The prototype LPxTG-containing protein has a signal sequence peptide located in the N-terminus and a sorting motif in the C-terminus. The signal sequence enables the precursor surface protein to be transported across the plasma membrane *via* protein secretion systems (Hendrickx et al., 2009). Furthermore, *srtA* (SGO_RS06040), encoding class A sortase, was up-regulated with a fold change of 1.99 as well. Class A sortase enzymes cleave the sorting motif in the C-terminus of precursor cell-wall proteins and help anchor LPxTG-containing proteins to the cell wall (Pucciarelli et al., 2005). The present study indicated that, in response to the dual-species coaggregation, *S. gordonii* might utilize the secA2 system and class A sortase enzymes to produce an increased number of cell wall-anchored proteins, enhancing *S. gordonii*’s adherence ability.

#### 4.1.2 Dual-Species Coaggregation Induced Changes in Sources of Energy of *S. gordonii*

Two primary sources of energy of *S. gordonii* are arginine deimination and sugar phosphorylation (Pessione, 2012; Jesionowski et al., 2016). The dual-species coaggregation induced significant transcriptional changes in genes associated with bacterial metabolism. The results indicated that the arginine deimination was turned down, and the carbohydrate utilization was enhanced in *S. gordonii*.

The arginine repressor is responsible for suppressing arginine deiminase pathways in *S. gordonii*, and the gene that encodes arginine repressor (SGO_RS03435) was up-regulated (1.58-fold change) (Jakubovics et al., 2015; Robinson et al., 2018). *arcA* (SGO_RS07805), encoding arginine deiminase, was down-regulated (-2.60-fold change). Meanwhile, three genes involved in arginine biosynthesis were down-regulated with fold changes of -2.27 to -3.45: *arcB* (SGO_RS07800) encodes ornithine carbamoyltransferase, *argH* (SGO_RS00870) encodes argininosuccinate lyase, and *argJ* (SGO_RS07680) encodes bifunctional glutamate N-acetyltransferase/amino-acid acetyltransferase (**Table S2**). Based on the transcriptome results, the pathways of arginine biosynthesis and metabolism were repressed in *S. gordonii* following coaggregation. Kaplan *et al.* revealed that *F. nucleatum* utilized an arginine-inhibitable adhesin (RadD) to adhere to *S. gordonii*, and the presence of arginine impaired the coaggregation between these two species (Kaplan et al., 2009). The present results indicated a possibility that *S. gordonii* repressed the pathway of arginine biosynthesis to reduce arginine production and maintain a symbiotic relationship with the attached *F. nucleatum* subsp*. polymorphum*. The reduced production of arginine by *S. gordonii* and the low abundance of arginine in the buffer environment might lead to the substrate shortage, making it difficult for *S. gordonii* to utilize the arginine deiminase pathway to store energy.

*S. gordonii* might thus switch to sugar phosphorylation and increase carbohydrate utilization to produce enough energy for itself. This hypothesis was supported by the present transcriptome results. Dozens of genes involved in carbohydrate metabolism were significantly up-regulated, and they were enriched in the pathways of PTS and starch and sucrose metabolism. The PTS comprises Enzyme I (EI), histidine phosphocarrier protein (HPr), and a group of substrate-specific Enzyme II (EII) complexes (Postma et al., 1993; Tong et al., 2011). The *S. gordonii* gene (SGO_RS07625) that encodes HPr was up-regulated with a fold change of 2.41 in response to *S. gordonii*-*F. nucleatum* subsp*. polymorphum* coaggregation. Seven more genes encoding PTS cellobiose/mannose/beta-glucoside transporter subunits were up-regulated with fold changes of at least two, increasing *S. gordonii*’s uptake of sugar (**Table S2**). The gene *glgB* (SGO_RS07615) and four contiguous genes in its downstream region (*glgC*, SGO_RS07610; *glgD*, SGO_RS07605; *glgA*, SGO_RS07600; and *glgP-1*, SGO_RS07595) were all involved in glycogen biosynthesis and significantly up-regulated (**Table S2**). Glycogen is readily metabolized and is considered a preferable form of stored energy source in bacteria, playing a crucial role in bacterial survival (Preiss, 1984). We further quantified intracellular glycogen content and confirmed that the glycogen content was significantly higher in coaggregates than in monocultures (**Figure S1**).

In addition, *ccpA* (SGO_RS01170) encoding *S. gordonii* catabolite control protein A (CcpA) was down-regulated with a fold change of -2.85 following dual-species coaggregation. Carbon catabolite repression (CCR) is essential for optimizing energy production in most bacteria (Görke and Stülke, 2008). During CCR, CcpA represses the metabolism of less preferred carbohydrate sources (Zheng et al., 2012). When rapidly metabolizable carbohydrates are abundant and available, genes associated with the utilization of less preferred carbohydrates are repressed (Deutscher et al., 2006; Deutscher, 2008). The downregulation of *ccpA* might lift CCR, encouraging *S. gordonii* cells to metabolize all available carbohydrates and store more energy.

### 4.2 Analysis of the Transcriptional Profiling of *F. nucleatum* subsp. *polymorphum*

The lack of efficient genetic and molecular systems has hindered the further study of *F. nucleatum* at the molecular level. Although shuttle plasmid pHS17 was constructed to study properties of *F. nucleatum* subsp. *polymorphum* (ATCC 10953), transformation efficiency of pHS17 was low (Haake et al., 2000; Kinder Haake et al., 2006). Only a limited number of studies constructed trait-specific isogenic mutants to validate *F. nucleatum* gene functions; hence there was insufficient research to aid the further transcriptome analysis of many *F. nucleatum* subsp. *polymorphum* DEGs.

Genes that encode ABC transporters were differentially regulated following coaggregation with *S. gordonii*. Five DEGs that encode different types of ABC transporters were identified (**Table S5**). Among them, genes AT688_RS00240 and AT688_RS00245 were significantly up-regulated, and they might have multiple functions such as participating in cell division, hemin transport, and acetoin utilization (Yoshida et al., 2000; Bouige et al., 2002; Friedman et al., 2006). The other three DEGs were significantly down-regulated with fold changes ranged from -2.14 to -4.20. One of them encodes LPS export system permeases (AT688_RS05820, LptF/LptG family permease), facilitating LPS assembly (Ruiz et al., 2008), and the downregulation of the gene might hinder the process of bacterial LPS assembly in *F. nucleatum* subsp. *polymorphum*.

### 4.3 Coaggregation Between *S. gordonii* and *F. nucleatum* subsp. *polymorphum* Promoted Bacterial Intracellular Survival and Attenuated Macrophages’ Inflammatory Responses

The innate immune system is actively against microbial pathogens. Macrophages are common innate immune cells that respond to microbial infection, recognize and engulf microbial pathogens (phagocytosis), and create an internal compartment where pathogens may be killed (phagosomes) (Gordon, 2016). Past research showed that bacterial species could use multiple strategies to survive within macrophages. For instance, pathogens could reduce immunogenicity to hide from macrophage defense, bond with inhibitory receptors to mitigate host immune responses, or subvert phagosome maturation (Sarantis and Grinstein, 2012; Bor et al., 2016; Mitchell et al., 2016; Thakur et al., 2019). Bor *et al.* discovered that the interspecies coaggregation between *Candida albicans* (*C. albicans*) and *F. nucleatum* attenuated both species’ virulence, contributing to a long-term commensal lifestyle and prolonged survival of both species within macrophages (Bor et al., 2016). To survive and even replicate within macrophages, *Candida glabrata* could mask immunostimulatory cell-wall components and induce poor host cell activation, causing low levels of pro-inflammatory cytokines upon infection (Kasper et al., 2015). *Mycobacterium tuberculosis* has learned to counter-balance the macrophage killing by reducing inflammasome activation and facilitating immune evasion of the bacterium (Weiss and Schaible, 2015).

The present study revealed the transcriptional profiling of *S. gordonii* and *F. nucleatum* subsp. *polymorphum* in response to the short-term dual-species coaggregation. Further studies are encouraged to investigate transcriptional changes of coaggregated species over a longer period to observe the trends of gene regulation in partner species. Nevertheless, in this study, 30 minutes of coaggregation between *S. gordonii* and *F. nucleatum* subsp. *polymorphum* has induced significant transcriptional changes in multiple DEGs related to the biosynthesis and export of peptidoglycan and LPS as well as carbohydrate and pyruvate metabolism. In addition, GC-MS analysis revealed that the coaggregates of *S. gordonii* and *F. nucleatum* subsp. *polymorphum* produced significantly reduced levels of propanoic acid and butyric acid compared with the co-culture of both species. Furthermore, the dual-species coaggregation group exhibited significantly better intracellular survival within macrophages and induced markedly lower production of IL-6 and IL-1β compared with the dual-species co-culture group. Transcriptome results together with GC-MS analysis indicated that both species following coaggregation are likely to resist macrophage killing *via* mitigation of the immune responses. Intergeneric coaggregation between *F. nucleatum* subsp. *polymorphum* and *S. gordonii* prepared both opportunistic pathogens for better resilience against macrophage killing.

#### 4.3.1 *S. gordonii* DEGs Associated With the Pyruvate Metabolism and Peptidoglycan Biosynthesis Were Affected Following Coaggregation

Transcriptome analysis revealed that *S. gordonii* increasingly metabolized pyruvate to stimulate the TCA cycle that releases more stored energy. Pyruvate dehydrogenase complex is a complex of three enzymes and converts pyruvate into acetyl-CoA which fuels the TCA cycle (DeBrosse and Kerr, 2016). In our study, *S. gordonii* genes encoding these three enzymes (SGO_RS05555, SGO_RS05560, and SGO_RS05570) were all up-regulated with fold changes of at least 2.0 following coaggregation. A gene that encodes alpha-ketoacid dehydrogenase subunit beta (SGO_RS05565) was also significantly up-regulated, suggesting that the TCA cycle activity was enhanced (**Table S2**).

Since pyruvate was mostly converted to primary products for the TCA cycle, less pyruvate was available for producing metabolites such as SCFAs. To evaluate the effect of dual-species coaggregation on the SCFAs secretion by *S. gordonii* and *F. nucleatum* subsp. *polymorphum*, we measured and compared the concentrations of SCFAs in supernatants of different culture groups. Results showed that, compared with the mono-species groups and dual-species co-culture group, the dual-species coaggregation group produced significantly decreased concentrations of propanoic acid and butyric acid. Results indicated that dual-species coaggregation greatly curtailed the overall production of propanoic acid and butyric acid. Since the propanoic acid and butyric acid levels were determined in culture media, whether the production pattern is similar inside macrophage requires further study. Several studies found that propionic acid and butyric acid stimulated pro-inflammatory responses of periodontal ligament cells and inhibited cell growth (Niederman et al., 1997a; Niederman et al., 1997b; Jeng et al., 1999; Mirmonsef et al., 2012). Previous studies showed rather conflicting evidence regarding the effects of propanoic acid and butyric acid on the inflammatory responses of macrophages (Al-Lahham et al., 2012; Tannahill et al., 2013; Schulthess et al., 2019). Nevertheless, Schulthess *et al.* demonstrated that butyric acid enhanced the antimicrobial function of macrophages as a consequence of glycolysis and mTOR inhibition. Macrophages treated with butyric acid exhibited increased killing of intracellular *Salmonella* (Schulthess et al., 2019).

Gene *murE* (SGO_RS08025), involved in the biosynthesis of cell-wall peptidoglycan, was down-regulated (-2.42-fold change) following coaggregation. Host immune cells, such as neutrophils and macrophages, could recognize bacterial peptidoglycan which is a prime pathogen-associated molecular pattern (PAMP), enhancing host immune and inflammatory responses (Wolf and Underhill, 2018).

Last but not least, it is a well-known fact that production of butyrate is an important virulence for *F. nucleatum*. *F. nucelatum* utilizes amino acids such as glutamate, lysine, and fructose as an energy source and produce SCFAs including butyrate (Himi et al., 2020; Llama-Palacios et al., 2020). Although we didn’t identify relevant DEGs in *F. nucelatum* subsp. *polymorphum*, the transcriptome results revealed that multiple *F. nucleatum* genes were of unknown functions, and the reduced production of butyrate might be facilitated by possibly novel metabolic pathways of *F. nucelatum* subsp. *polymorphum*.

#### 4.3.2 *F. nucleatum* subsp. *polymorphum* DEGs Associated With the Biosynthesis of LPS and Peptidoglycan Were Down-Regulated

LPS is a common outer membrane component of *F. nucleatum*, and it serves as a potent PAMP that activates macrophages and induces host pro-inflammatory responses (Fujihara et al., 2003). Glycosyltransferase family 9 proteins (GT9 proteins) play an essential role in bacterial virulence since they consist of enzymes such as heptosyltransferase, which is thought to be involved in the biosynthesis of LPS (Kadrmas and Raetz, 1998; Gronow et al., 2000). In response to coaggregation, two genes encoding GT9 proteins were down-regulated (AT688_RS08670, lipopolysaccharide heptosyltransferase family, -1.80-fold change; AT688_RS04635, ADP-heptose–LPS heptosyltransferase, -1.92-fold change). ADP-heptose:LPS heptosyltransferase is an enzyme of LPS inner core region biosynthesis. Pfannkuch et al. revealed that ADP heptose acted as a general bacterial PAMP, and it was more potent than mature LPS in activating NF-κB signaling in epithelial cells (Pfannkuch et al., 2019). Macrophages orchestrate host innate immune responses to bacterial LPS by expressing various inflammatory cytokines, including IL-6, IL-1β, and TNF-α (Guha and Mackman, 2001). In addition, DEG (AT688_RS01820) involved in the peptidoglycan biosynthesis process were down-regulated with a fold change of -2.03. The downregulation of genes related to LPS or peptidoglycan biosynthesis might reduce the immunogenicity of *F. nucleatum* subsp. *polymorphum*, serving as a strategy for mitigating host immune responses and improving bacterial survival within macrophages. However, direct evidence showing that coaggregation reduce LPS production is still lacking. We tried several methods to isolate LPS from the coaggregated pellets, but we failed to extract pure LPS for quantification, probably due to the interference of lipoteichoic acid that shares similar structures with LPS.

Co-culture experiments of macrophages and bacterial cells revealed that coaggregation promoted the intracellular survival rates of *F. nucleatum* subsp. *polymorphum* and *S. gordonii* and reduced the secretion of pro-inflammatory cytokines by macrophages. The mutual attenuation of virulence of coaggregated *F. nucleatum* subsp. *polymorphum* and *S. gordonii* towards macrophages may be attributable to the reduced production of propanoic acid and butyric acid by coaggregated species and the downregulation of the above-mentioned DEGs involved in the biosynthesis and transport of peptidoglycan and LPS. The phagosome maturation and generation of reactive oxygen species also play a crucial role in bactericidal killing, and multiple bacterial species were capable of tackling these processes (Sarantis and Grinstein, 2012). Croft et al. found that *S. gordonii* with endocarditis pathogenic potential could resist phagosome killing in macrophages by promoting autophagy or subverting phagosome maturation (Croft et al., 2018). Salvatori et al. revealed that *S. gordonii* promoted greater escape of *C. albicans* from macrophages without affecting phagosome maturation (Salvatori et al., 2020). The transcriptome results of *F. nucleatum* subsp. *polymorphum* revealed that *ahpC*, encoding peroxiredoxin, was significantly up-regulated following coaggregation (AT688_RS00700, 2.99-fold change). The *ahpC*-encoding protein catalyzes the reduction of hydrogen peroxide and organic hydroperoxides and thus protects cells against oxidative stress induced by macrophages (Poole, 2005). Previous research uncovered that mutation of *crp*, an activator of *ahpC*, led to a reduced intracellular survival rate of *Mycobacterium tuberculosis* within macrophages (Rickman et al., 2005; Lee et al., 2014). The upregulation of *ahpC* following *S. gordonii-F. nucleatum* subsp. *polymorphum* coaggreagtion might render bacteria resistant against reactive oxygen species generated by macrophages.

## 5 Conclusions

Interspecies coaggregation between *S. gordonii* and *F. nucleatum* subsp. *polymorphum* resembles the *in vivo* bacterial interplay in the oral multispecies community. The present study revealed that interspecies coaggregation altered transcriptional profiling of *S. gordonii* and *F. nucleatum* subsp. *polymorphum*, promoted symbiotic bacterial survival within macrophages, and attenuated macrophages’ inflammatory responses. More rigorously designed studies are warranted further investigate the specific roles of candidate DEGs in modifying bacterial pathogenicity.

## Data Availability Statement

The datasets presented in this study can be found in online repositories. The names of the repository/repositories and accession number(s) can be found below: https://www.ncbi.nlm.nih.gov/geo/, GSE164282.

## Author Contributions

LG and TL designed and conducted experiments. LG and TL analyzed the data. TL and RY wrote the manuscript. All authors contributed to the article and approved the submitted version.

## Funding

This research was funded by a grant from the National Natural Science Foundation of China to LG (number: 81670982).

## Conflict of Interest

The authors declare that the research was conducted in the absence of any commercial or financial relationships that could be construed as a potential conflict of interest.

## Publisher’s Note

All claims expressed in this article are solely those of the authors and do not necessarily represent those of their affiliated organizations, or those of the publisher, the editors and the reviewers. Any product that may be evaluated in this article, or claim that may be made by its manufacturer, is not guaranteed or endorsed by the publisher.

## Abbreviations

BHI, Brain Heart Infusion; CAB, coaggregation buffer; CCK-8, Cell Counting Kit-8; CFU, colony-forming units; DEG, differentially expressed genes; dTHP-1 cells, THP-1 derived macrophages; ELISA, Enzyme-Linked Immunosorbent Assay; *F. nucleatum*, *Fusobacterium nucleatum*; FBS, fetal bovine serum; FDR, false discovery rate; GC-MS, gas chromatography-mass spectrometry; GO, Gene Ontology; GT9 proteins, glycosyltransferase family 9 proteins; HPr, histidine phosphocarrier protein; KEGG, Kyoto Encyclopedia of Genes and Genomes; LPS, lipopolysaccharide; LPxTG, Leu-Pro-x-Thr-Gly; MOI, multiplicity of infection; OD, optical density; PAMP, pathogen-associated molecular pattern; PPI, Protein-Protein Interaction; PTS, phosphotransferase system; *S. gordonii*, *Streptococcus gordonii*; RT-qPCR, quantitative reverse transcription PCR; SCFAs, short-chain fatty acids; TCA cycle, the citrate cycle.
