# Supplementary material for: Interactions Between Streptococcus gordonii and Fusobacterium nucleatum Altered Bacterial Transcriptional Profiling and Attenuated the Immune Responses of Macrophages
Source: Front Cell Infect Microbiol. 2022 Jan 7;11:783323. doi: 10.3389/fcimb.2021.783323 (PMC8776643; doi:10.3389/fcimb.2021.783323)
Supplement: Supplementary file 1 [file DataSheet_1.pdf]

**Tables**

**Table S1. PCR primers**

| Locus ID | Sequence (5’-3’) | Product (bp) |
|---|---|---|
| ***S. gordonii* DL1** | | |
| 16S rRNA | F: AGACACGGCCCAGACTCCTAC | 137 |
| | R: TCACACCCGTTCTTCTCTTACAA | |
| SGO_RS01220 | F: GATGTTCGTCGTCCTGCCTT | 114 |
| | R: TGGCACACGTCCAAGAGAAG | |
| SGO_RS05565 | F: TGGCGAAATCAAACGCGAAG | 142 |
| | R: AAGCGGAACTAGGGTACGTG | |
| SGO_RS07615 | F: TGGGCTACTTCGCCTTTGAG | 165 |
| | R: CGAAAGTCGGTGTCCCATCA | |
| SGO_RS07625 | F: ACAGGTATTCACGCTCGTCC | 152 |
| | R: ACTACGTCTGCACCTTGACC | |
| SGO_RS07800 | F: AGATGCTGACGAAGCTGTGA | 183 |
| | R: TGGAAAGCAGGCAAGCAGT | |
| ***F. nucleatum* subsp. *polymorphum* ATCC 10953** | | |
| 16S rRNA | F: AGCGCGTCTAGGTGGTTATG | 153 |
| | R: CCCCATCGGCATTCCTACAA | |
| AT688_RS00240 | F: ATCAACTCCAAGTGCCCAAGC | 90 |
| | R: AATCCTGCACTAGCTCCATCTG | |
| AT688_RS03075 | F: AGCCCTGAGGAAAGAGCAA | 135 |
| | R: AAATGGACTTTCATCTCTTGTGCT | |
| AT688_RS11260 | F: AAAATAGAGGAGTTGCTTTTGGACT | 168 |
| | R: TTCCTACTGCTCCTGCAAATATCA | |

**Table S2. Key *S. gordonii* genes that were significantly regulated following coaggregation with *F. nucleatum* subsp. *polymorphum*.**

| Locus tag | Gene description | Fold change | *P* adj | Gene symbol /old locus tag |
|---|---|---|---|---|
| **Arginine biosynthesis and metabolism** | | | | |
| SGO_RS07800 | ornithine carbamoyltransferase | -3.45 | <0.01 | *argF*, *arcB* SGO_1592 |
| SGO_RS00870 | argininosuccinate lyase | -2.27 | <0.01 | *argH* SGO_0176 |
| SGO_RS07680 | bifunctional glutamate N-acetyltransferase/amino-acid acetyltransferase ArgJ | -3.26 | <0.01 | *argJ* SGO_1568 |
| SGO_RS07805 | arginine deiminase | -2.60 | <0.01 | *arcA* SGO_1593 |
| **ATP binding** | | | | |
| SGO_RS02520 | primosomal protein DnaI | 2.27 | <0.01 | *dnaI* SGO_0510 |
| **ATP-binding cassette (ABC) transporter** | | | | |
| SGO_RS01985 | ABC transporter ATP-binding protein | 2.41 | <0.01 | SGO_0398 |
| SGO_RS01990 | ABC transporter permease | 2.27 | <0.01 | SGO_0399 |
| SGO_RS05200 | phosphate ABC transporter ATP-binding protein | 2.02 | <0.01 | *pstB* SGO_1059 |
| SGO_RS01485 | ATP-binding cassette domain-containing protein | -2.48 | <0.01 | SGO_0301 |
| SGO_RS01495 | ATP-binding cassette domain-containing protein | -2.21 | <0.01 | SGO_0303 |
| SGO_RS01505 | ABC transporter permease | -2.77 | <0.01 | SGO_0305 |
| SGO_RS01510 | ATP-binding cassette domain-containing protein | -3.28 | <0.01 | SGO_0306 |
| SGO_RS04620 | ABC transporter ATP-binding protein | -2.28 | <0.01 | SGO_0944 |
| SGO_RS06395 | sugar ABC transporter permease | -2.20 | <0.01 | SGO_1304 |
| SGO_RS06955 | ABC transporter permease | -6.11 | <0.01 | SGO_1418 |
| SGO_RS06960 | ABC transporter ATP-binding protein | -2.84 | 0.01 | SGO_1419 |
| SGO_RS08585 | ABC transporter ATP-binding protein | -2.63 | <0.01 | SGO_1751 |
| SGO_RS08655 | sugar ABC transporter permease | -2.16 | <0.01 | SGO_1765 |
| SGO_RS10260 | peptide cleavage/export ABC transporter | -12.91 | <0.01 | SGO_2097 |
| **Biosynthesis of cell-wall peptidoglycan** | | | | |
| SGO_RS08025 | UDP-N-acetylmuramoyl-L-alanyl-D-glutamate--L-lysine ligase | -2.42 | <0.01 | *murE* SGO_1638 |
| **Carbohydrate derivative metabolism** | | | | |
| SGO_RS04960 | dTDP-glucose 4,6-dehydratase | 3.14 | <0.01 | *rfbB-1* SGO_1011 |

| | | | | |
|---|---|---|---|---|
| SGO_RS10915 | phosphopentomutase | 2.21 | <0.01 | *deoB* SGO_1264 |
| SGO_RS10920 | ribose-5-phosphate isomerase RpiA | 2.01 | <0.01 | *rpiA* SGO_1265 |
| SGO_RS06190 | purine-nucleoside phosphorylase | 2.20 | <0.01 | *deoD* SGO_1260 |
| **Carbohydrate metabolism** | | | | |
| SGO_RS00495 | type I pullulanase | 2.20 | <0.01 | *pulA* SGO_0099 |
| SGO_RS01220 | formate C-acetyltransferase | 2.22 | <0.01 | *pflB* SGO_0247 |
| SGO_RS04370 | phosphoglucosamine mutase | 2.32 | <0.01 | *glmM* SGO_0889 |
| SGO_RS04965 | UDP-glucose 4-epimerase GalE | 2.54 | <0.01 | *galE-1* SGO_1012 |
| SGO_RS07595 | glycogen/starch/alpha-glucan phosphorylase | 2.53 | <0.01 | *glgP-1* SGO_1550 |
| SGO_RS04955 | dTDP-4-dehydrorhamnose 3,5-epimerase family protein | 3.04 | <0.01 | *rmlC* SGO_1010 |
| SGO_RS04950 | glucose-1-phosphate thymidylyltransferase RfbA | 2.76 | <0.01 | *rfbA-1* SGO_1009 |
| SGO_RS07600 | glycogen synthase GlgA | 2.99 | <0.01 | *glgA* SGO_1551 |
| SGO_RS07605 | glucose-1-phosphate adenylyltransferase subunit GlgD | 2.63 | <0.01 | *glgD* SGO_1552 |
| SGO_RS07610 | glucose-1-phosphate adenylyltransferase | 2.92 | <0.01 | *glgC* SGO_1553 |
| SGO_RS07615 | 1,4-alpha-glucan branching protein GlgB | 3.18 | <0.01 | *glgB* SGO_1554 |
| SGO_RS09100 | sucrose-6-phosphate hydrolase | -2.45 | <0.01 | SGO_1858 |
| SGO_RS08770 | glycyl radical protein | -2.22 | <0.01 | SGO_1788 |
| **Competence protein** | | | | |
| SGO_RS09415 | type II secretion system F family protein | -5.92 | <0.01 | *comYB* SGO_1923 |
| SGO_RS09420 | competence protein CglA | -2.97 | <0.01 | *comYA* SGO_1924 |
| **Fatty acid biosynthesis** | | | | |
| SGO_RS08305 | 3-oxoacyl-[acyl-carrier-protein] reductase | -2.21 | <0.01 | *fabG* SGO_1693 |
| SGO_RS08310 | ACP S-malonyltransferase | -2.33 | <0.01 | *fabD* SGO_1694 |
| SGO_RS08325 | ketoacyl-ACP synthase III | -2.17 | <0.01 | *fabH* SGO_1698 |
| SGO_RS08335 | enoyl-CoA hydratase | -3.08 | <0.01 | SGO_1700 |
| SGO_RS08320 | acyl carrier protein | -2.37 | <0.01 | *acp* SGO_1697 |
| **Glycosyltransferase** | | | | |
| SGO_RS00525 | 4-alpha-glucanotransferase | -2.36 | <0.01 | *malQ* SGO_0105 |
| **Hydrolase activity** | | | | |
| SGO_RS04410 | alpha/beta hydrolase | -3.00 | <0.01 | SGO_0899 |
| SGO_RS01175 | serine hydrolase | -2.77 | <0.01 | SGO_0238 |
| **Membrane or cell wall- associated proteins** | | | | |
| SGO_RS09810 | isopeptide-forming domain-containing fimbrial protein | 5.42 | <0.01 | SGO_2005 |

| | | | | |
|---|---|---|---|---|
| SGO_RS09805 | LPXTG cell wall anchor domain-containing protein | 3.23 | <0.01 | SGO_2004 |
| SGO_RS02365 | cell wall-binding protein | 2.59 | <0.01 | SGO_0478 |
| SGO_RS00535 | LPXTG cell wall anchor domain-containing protein | 2.53 | <0.01 | SGO_0107 |
| SGO_RS04150 | cell wall protein | 3.44 | <0.01 | SGO_0846 |
| **Nucleic acid metabolism** | | | | |
| SGO_RS00755 | nucleoside deaminase | -2.34 | 0.01 | SGO_0153 |
| **Nucleotide metabolism** | | | | |
| SGO_RS01365 | dihydroorotate oxidase | -3.31 | <0.01 | *pyrA* SGO_0277 |
| SGO_RS04655 | uridine kinase | -2.08 | 0.01 | *udk* SGO_0951 |
| SGO_RS09735 | adenylosuccinate synthetase | -2.34 | <0.01 | *purA* SGO_1989 |
| **Oxidoreductase activity** | | | | |
| SGO_RS00610 | Gfo/Idh/MocA family oxidoreductase | 2.30 | <0.01 | SGO_0123 |
| SGO_RS01660 | thioredoxin | 2.67 | <0.01 | *trxA* SGO_0337 |
| SGO_RS02525 | NADPH-dependent oxidoreductase | 2.41 | <0.01 | SGO_0511 |
| SGO_RS04095 | nitroreductase family protein | 2.38 | <0.01 | SGO_0835 |
| SGO_RS04650 | Gfo/Idh/MocA family oxidoreductase | 2.04 | <0.01 | SGO_0950 |
| SGO_RS05745 | FAD-dependent oxidoreductase | 2.49 | <0.01 | SGO_1167 |
| SGO_RS05765 | thiol-disulfide oxidoreductase-associated lipoprotein SdbB | 2.23 | <0.01 | *sdbB* SGO_1171 |
| SGO_RS09815 | TlpA family protein disulfide reductase | 6.52 | <0.01 | SGO_2006 |
| SGO_RS08315 | enoyl-[acyl-carrier-protein] reductase FabK | -2.62 | <0.01 | SGO_1695 |
| **Phosphoenolpyruvate-dependent sugar phosphotransferase system (PTS)** | | | | |
| SGO_RS07625 | phosphocarrier protein HPr | 2.41 | <0.01 | SGO_1556 |
| SGO_RS07720 | PTS cellobiose transporter subunit IIC | 2.03 | <0.01 | *ptcC* SGO_1576 |
| SGO_RS07730 | PTS cellobiose transporter subunit IIA | 2.80 | <0.01 | SGO_1578 |
| SGO_RS07740 | PTS cellobiose transporter subunit IIB | 2.22 | 0.01 | SGO_1580 |
| SGO_RS08235 | PTS mannose transporter subunit IIAB | 2.05 | <0.01 | *manX* SGO_1679 |
| SGO_RS08240 | PTS mannose/fructose/sorbose transporter subunit IIC | 2.06 | <0.01 | SGO_1680 |
| SGO_RS08245 | PTS mannose/fructose/sorbose transporter family subunit IID | 2.17 | <0.01 | SGO_1681 |
| SGO_RS01385 | PTS glucose transporter subunit IIA | 2.00 | <0.01 | SGO_0281 |
| SGO_RS09095 | PTS beta-glucoside transporter subunit IIBCA | -3.32 | <0.01 | SGO_1857 |

| | | | | |
|---|---|---|---|---|
| SGO_RS09270 | PTS fructose transporter subunit IIA | -2.27 | 0.01 | SGO_1893 |
| **Protein metabolism** | | | | |
| SGO_RS02345 | CPBP family intramembrane metalloprotease | 5.00 | <0.01 | SGO_0474 |
| SGO_RS04100 | dipeptidase PepV | 2.51 | <0.01 | *pepV* SGO_0836 |
| SGO_RS07645 | CPBP family intramembrane metalloprotease | 14.62 | <0.01 | SGO_1560 |
| SGO_RS07995 | ATP-dependent Clp protease proteolytic subunit | 2.22 | <0.01 | *clpP* SGO_1632 |
| SGO_RS08925 | CPBP family intramembrane metalloprotease | -2.37 | <0.01 | SGO_1819 |
| **Protein transport** | | | | |
| SGO_RS04770 | accessory Sec system protein Asp3 | 2.67 | <0.01 | *asp3* SGO_0973 |
| **Pyruvate metabolism** | | | | |
| SGO_RS05555 | dihydrolipoyl dehydrogenase | 2.51 | <0.01 | *lpdA* SGO_1130 |
| SGO_RS05560 | dihydrolipoamide acetyltransferase | 2.40 | <0.01 | *sucB* SGO_1131 |
| SGO_RS05565 | alpha-ketoacid dehydrogenase subunit beta | 2.44 | <0.01 | SGO_1132 |
| SGO_RS05570 | thiamine pyrophosphate-dependent dehydrogenase E1 component subunit alpha | 2.05 | <0.01 | *acoA* SGO_1133 |
| **Transcription** | | | | |
| SGO_RS00500 | LacI family transcriptional regulator | 2.11 | <0.01 | SGO_0100 |
| SGO_RS01970 | helix-turn-helix transcriptional regulator | 3.30 | <0.01 | SGO_0395 |
| SGO_RS02455 | helix-turn-helix domain-containing protein | 2.64 | <0.01 | *rgg* SGO_0496 |
| SGO_RS02815 | helix-turn-helix transcriptional regulator | 5.69 | <0.01 | SGO_0572 |
| SGO_RS07660 | helix-turn-helix transcriptional regulator | 13.93 | <0.01 | SGO_1564 |
| SGO_RS09430 | DNA-directed RNA polymerase subunit beta | 3.01 | <0.01 | *rpoC* SGO_1926 |
| SGO_RS09435 | DNA-directed RNA polymerase subunit beta | 3.22 | <0.01 | *rpoB* SGO_1927 |
| SGO_RS09195 | TetR/AcrR family transcriptional regulator | -2.64 | <0.01 | SGO_1878 |
| SGO_RS00630 | MurR/RpiR family transcriptional regulator | -2.51 | <0.01 | SGO_0127 |
| SGO_RS01170 | CcpA protein (Proteinase) | -2.85 | <0.01 | *ccpA* SGO_0237 |
| SGO_RS01840 | LysR family transcriptional regulator | -2.23 | <0.01 | SGO_0370 |
| SGO_RS01845 | helix-turn-helix transcriptional regulator | -2.90 | <0.01 | SGO_0371 |

| | | | | |
|---|---|---|---|---|
| SGO_RS02250 | YebC/PmpR family DNA-binding transcriptional regulator | -2.45 | <0.01 | SGO_0454 |
| SGO_RS04405 | helix-turn-helix domain-containing protein | -5.23 | <0.01 | SGO_0898 |
| SGO_RS04475 | MarR family transcriptional regulator | -3.07 | <0.01 | SGO_0912 |
| SGO_RS05505 | GntR family transcriptional regulator | -3.10 | <0.01 | SGO_1121 |
| SGO_RS06660 | Rgg/GadR/MutR family transcriptional regulator | -2.86 | <0.01 | *rggB* SGO_1359 |
| SGO_RS08330 | MarR family transcriptional regulator | -2.23 | <0.01 | SGO_1699 |
| SGO_RS08590 | MarR family transcriptional regulator | -2.93 | <0.01 | SGO_1752 |
| SGO_RS08980 | aspartate aminotransferase | -4.48 | <0.01 | SGO_1830 |
| SGO_RS10000 | helix-turn-helix transcriptional regulator | -2.54 | <0.01 | SGO_2047 |
| SGO_RS03115 | hypothetical protein | -2.17 | <0.01 | SGO_0633 |
| SGO_RS00990 | pur operon repressor | -2.71 | <0.01 | *purR* SGO_0202 |

**Table S3. *S. gordonii* DEGs enriched in key GO biological processes.**

| Locus tag | Gene description | Fold change | *P* adj | Gene symbol /old locus tag |
|---|---|---|---|---|
| **Ten DEGs involved in six interrelated biological processes** | | | | |
| SGO_RS02810 | DUF3278 domain-containing protein | 3.56 | <0.01 | SGO_0571 |
| SGO_RS07610 | glucose-1-phosphate adenylyltransferase | 2.92 | <0.01 | *glgC* SGO_1553 |
| SGO_RS07615 | 1,4-alpha-glucan branching protein GlgB | 3.18 | <0.01 | *glgB* SGO_1554 |
| SGO_RS04960 | dTDP-glucose 4,6-dehydratase | 3.14 | <0.01 | *rfbB-1* SGO_1011 |
| SGO_RS04965 | UDP-glucose 4-epimerase GalE | 2.54 | <0.01 | *galE-1* SGO_1012 |
| SGO_RS00560 | acyltransferase | 2.37 | <0.01 | SGO_0112 |
| SGO_RS04950 | glucose-1-phosphate thymidylyltransferase RfbA | 2.76 | <0.01 | *rfbA-1* SGO_1009 |
| SGO_RS04955 | dTDP-4-dehydrorhamnose 3,5-epimerase family protein | 3.04 | <0.01 | *rmlC* SGO_1010 |
| SGO_RS07595 | glycogen/starch/alpha-glucan phosphorylase | 2.53 | <0.01 | *glgP-1* SGO_1550 |
| SGO_RS08320 | acyl carrier protein | -2.37 | <0.01 | *ACP* SGO_1697 |
| **Arginine biosynthesis and metabolism** | | | | |
| SGO_RS07800 | ornithine carbamoyltransferase | -3.45 | <0.01 | *argF*, *arcB* SGO_1592 |
| SGO_RS00870 | argininosuccinate lyase | -2.27 | <0.01 | *argH* SGO_0176 |
| SGO_RS07680 | bifunctional glutamate N-acetyltransferase/ amino-acid acetyltransferase ArgJ | -3.26 | <0.01 | *argJ* SGO_1568 |
| SGO_RS07805 | arginine deiminase | -2.60 | <0.01 | *arcA* SGO_1593 |

**Table S4.** ***S. gordonii*** **DEGs enriched in key KEGG pathways.**

| Locus tag | Gene description | Fold change | *P* adj | Gene symbol /old locus tag |
|---|---|---|---|---|
| **Fatty acid biosynthesis** | | | | |
| SGO_RS08305 | 3-oxoacyl-[acyl-carrier-protein] reductase | -2.21 | <0.01 | *fabG* SGO_1693 |
| SGO_RS08310 | ACP S-malonyltransferase | -2.33 | <0.01 | *fabD* SGO_1694 |
| SGO_RS08325 | ketoacyl-ACP synthase III | -2.17 | <0.01 | *fabH* SGO_1698 |
| SGO_RS08335 | enoyl-CoA hydratase | -3.08 | <0.01 | SGO_1700 |
| SGO_RS08315 | enoyl-[acyl-carrier-protein] reductase FabK | -2.62 | <0.01 | SGO_1695 |
| **Phosphotransferase system (PTS)** | | | | |
| SGO_RS01385 | PTS glucose transporter subunit IIA | 2.00 | <0.01 | SGO_0281 |
| SGO_RS07720 | PTS cellobiose transporter subunit IIC | 2.03 | <0.01 | *ptcC* SGO_1576 |
| SGO_RS07730 | PTS cellobiose transporter subunit IIA | 2.80 | <0.01 | SGO_1578 |
| SGO_RS07740 | PTS cellobiose transporter subunit IIB | 2.22 | 0.01 | SGO_1580 |
| SGO_RS08235 | PTS mannose transporter subunit IIAB | 2.05 | <0.01 | *manX* SGO_1679 |
| SGO_RS08240 | PTS mannose/fructose/sorbose transporter subunit IIC | 2.06 | <0.01 | SGO_1680 |
| SGO_RS08245 | PTS mannose/fructose/sorbose transporter family subunit IID | 2.17 | <0.01 | SGO_1681 |
| SGO_RS09270 | PTS fructose transporter subunit IIA | -2.27 | 0.01 | SGO_1893 |
| SGO_RS09095 | PTS beta-glucoside transporter subunit IIBCA | -3.32 | <0.01 | SGO_1857 |
| **Pyruvate metabolism** | | | | |
| SGO_RS05555 | dihydrolipoyl dehydrogenase | 2.51 | <0.01 | *lpdA* SGO_1130 |
| SGO_RS05560 | dihydrolipoamide acetyltransferase | 2.40 | <0.01 | *sucB* SGO_1131 |
| SGO_RS05565 | alpha-ketoacid dehydrogenase subunit beta | 2.44 | <0.01 | SGO_1132 |
| SGO_RS05570 | thiamine pyrophosphate-dependent dehydrogenase E1 component subunit alpha | 2.05 | <0.01 | *acoA* SGO_1133 |
| **Starch and sucrose metabolism** | | | | |
| SGO_RS07595 | glycogen/starch/alpha-glucan phosphorylase | 2.53 | <0.01 | *glgP-1* SGO_1550 |
| SGO_RS07600 | glycogen synthase GlgA | 2.99 | <0.01 | *glgA* SGO_1551 |
| SGO_RS07605 | glucose-1-phosphate adenylyltransferase subunit GlgD | 2.63 | <0.01 | *glgD* SGO_1552 |
| SGO_RS07610 | glucose-1-phosphate adenylyltransferase | 2.92 | <0.01 | *glgC* SGO_1553 |
| SGO_RS07615 | 1,4-alpha-glucan branching protein GlgB | 3.18 | <0.01 | *glgB* SGO_1554 |

| | | | | |
|---|---|---|---|---|
| SGO_RS00525 | 4-alpha-glucanotransferase | -2.36 | <0.01 | *malQ*<br>SGO_0105 |
| SGO_RS09100 | sucrose-6-phosphate hydrolase | -2.45 | <0.01 | SGO_1858 |

**Table S5. Key *F. nucleatum* subsp. *polymorphum* genes that were significantly regulated in response to coaggregation with *S. gordonii*.**

| Locus tag | Protein ID | Gene description | Fold change | *P* adj | Gene symbol/ old locus tag |
|---|---|---|---|---|---|
| **ABC transporter** | | | | | |
| AT688_RS00240 | WP_005895014.1 | ABC transporter substrate-binding protein | 13.51 | <0.01 | FNP_2382 |
| AT688_RS00245 | WP_005895008.1 | ABC transporter permease | 5.04 | 0.02 | FNP_2380 |
| AT688_RS06655 | WP_005898098.1 | ATP-binding cassette domain-containing protein | -2.89 | 0.04 | FNP_1554 |
| AT688_RS06650 | WP_005898100.1 | ABC transporter permease | -4.20 | <0.01 | FNP_1555 |
| AT688_RS05820 | WP_005896767.1 | LptF/LptG family permease | -2.14 | <0.01 | FNP_0780 |
| **Catalytic activity** | | | | | |
| AT688_RS06485 | WP_005897056.1 | histidine phosphatase family protein | -2.34 | <0.01 | FNP_0915 |
| **DNA repair** | | | | | |
| AT688_RS11400 | WP_005895357.1 | transcription-repair coupling factor | -2.16 | 0.02 | *mfd* FNP_0089 |
| AT688_RS06555 | WP_005897082.1 | uracil-DNA glycosylase | -9.55 | 0.01 | FNP_0930 |
| **Histidinol-phosphate transaminase activity** | | | | | |
| AT688_RS06180 | WP_005896915.1 | threonine-phosphate decarboxylase | -3.03 | <0.01 | FNP_0852 |
| **lipid metabolism** | | | | | |
| AT688_RS11035 | WP_005894657.1 | phosphatidylglycerophosphatase A | -3.55 | 0.03 | FNP_2231 |
| **Nucleic acid metabolism** | | | | | |
| AT688_RS07785 | WP_058229299.1 | restriction endonuclease subunit S | -2.12 | 0.02 | |
| AT688_RS07205 | WP_005897928.1 | rRNA pseudouridine synthase | -2.19 | 0.01 | FNP_1440 |
| AT688_RS03615 | WP_005895837.1 | ribosomal RNA small subunit methyltransferase A | -2.22 | <0.01 | *rsmA* FNP_0322 |
| AT688_RS07790 | WP_058229300.1 | restriction endonuclease subunit S | -2.32 | 0.01 | |
| AT688_RS10315 | WP_058229309.1 | restriction endonuclease subunit S | -2.34 | 0.02 | |
| AT688_RS03600 | WP_005895834.1 | 16S rRNA processing protein RimM | -5.26 | <0.01 | *rimM* FNP_0319 |
| **Oxidoreductase activity** | | | | | |
| AT688_RS06565 | WP_005897084.1 | SDR family NAD(P)-dependent oxidoreductase | -2.03 | 0.03 | FNP_0932 |
| AT688_RS03075 | WP_147373404.1 | peptide-methionine (R)-S-oxide reductase MsrB | 6.54 | 0.04 | *msrB* |
| AT688_RS00700 | WP_005894784.1 | peroxiredoxin | 2.99 | <0.01 | *ahpC* FNP_2288 |
| **Peptidoglycan synthesis** | | | | | |
| AT688_RS01820 | WP_005897403.1 | undecaprenyl-diphosphate phosphatase | -2.03 | <0.01 | FNP_1118 |
| **Protein export** | | | | | |
| AT688_RS11260 | WP_005895436.1 | lipoprotein signal peptidase | -3.55 | <0.01 | *lspA* FNP_0120 |
| AT688_RS02140 | WP_005897274.1 | preprotein translocase subunit SecY | 2.56 | <0.01 | *secY* FNP_1051 |
| AT688_RS04215 | WP_005896104.1 | preprotein translocase subunit YajC | 3.45 | <0.01 | *yajC* FNP_0448 |
| AT688_RS08695 | WP_005898423.1 | preprotein translocase subunit SecG | 2.20 | <0.01 | *secG* FNP_1802 |
| **Signal peptide processing** | | | | | |
| AT688_RS06685 | WP_005898086.1 | signal peptide peptidase SppA | -2.08 | 0.04 | *sppA* FNP_1548 |
| **Thiamine pyrophosphate binding** | | | | | |

| | | | | | |
|---|---|---|---|---|---|
| AT688_RS01705 | WP_005897443.1 | thiamine pyrophosphate-binding protein | -2.10 | <0.01 | FNP_1142 |
| **Transcription** | | | | | |
| AT688_RS07860 | WP_005897748.1 | RNA polymerase factor sigma-54 | -2.06 | <0.05 | *rpoN* FNP_1300 |
| AT688_RS10320 | WP_005898146.1 | putative DNA binding domain-containing protein | -2.27 | 0.02 | FNP_1591 |
| **Transferase activity** | | | | | |
| AT688_RS00365 | WP_005894949.1 | GNAT family N-acetyltransferase | -11.05 | <0.01 | FNP_2354 |
| AT688_RS05370 | WP_005896561.1 | GNAT family N-acetyltransferase | -2.68 | 0.04 | FNP_0687 |
| AT688_RS04675 | WP_005896285.1 | glycosyl transferase | -2.96 | 0.01 | FNP_0541 |
| AT688_RS08675 | WP_005898431.1 | glycosyltransferase family 2 protein | -2.05 | <0.05 | FNP_1806 |
| AT688_RS03620 | WP_005895838.1 | hypoxanthine phosphoribosyltransferase | -2.45 | <0.01 | *hpt* FNP_0323 |
| **Translation** | | | | | |
| AT688_RS00740 | WP_005894768.1 | 30S ribosomal protein S15 | 2.12 | <0.01 | *rpsO* FNP_2280 |
| AT688_RS02045 | WP_005897305.1 | 50S ribosomal protein L4 | 2.03 | <0.01 | *rplD* FNP_1070 |
| AT688_RS02050 | WP_005892379.1 | 50S ribosomal protein L23 | 2.03 | <0.01 | *rplW* FNP_1069 |
| AT688_RS02055 | WP_005897303.1 | 50S ribosomal protein L2 | 2.35 | <0.01 | *rplB* FNP_1068 |
| AT688_RS02075 | WP_005897296.1 | 50S ribosomal protein L16 | 2.27 | <0.01 | *rplP* FNP_1064 |
| AT688_RS02100 | WP_005897289.1 | 50S ribosomal protein L5 | 2.26 | <0.01 | *rplE* FNP_1059 |
| AT688_RS02125 | WP_005897278.1 | 30S ribosomal protein S5 | 2.41 | <0.01 | *rpsE* FNP_1054 |
| AT688_RS02130 | WP_005892817.1 | 50S ribosomal protein L30 | 2.33 | <0.01 | *rpmD* FNP_1053 |
| AT688_RS09940 | WP_005898763.1 | 30S ribosomal protein S16 | 2.61 | <0.01 | *rpsP* FNP_2060 |
| AT688_RS03845 | WP_005895940.1 | 50S ribosomal protein L20 | 2.61 | <0.01 | *rplT* FNP_0368 |
| AT688_RS05305 | WP_005896534.1 | 50S ribosomal protein L27 | 2.48 | <0.01 | *rpmA* FNP_0673 |
| AT688_RS09430 | WP_005898626.1 | 50S ribosomal protein L19 | 4.89 | <0.01 | *rplS* FNP_1955 |
| AT688_RS09690 | WP_005898701.1 | 50S ribosomal protein L32 | 2.69 | <0.01 | *rpmF* FNP_2008 |
| AT688_RS02005 | WP_005897324.1 | 30S ribosomal protein S18 | 2.71 | <0.01 | *rpsR* FNP_1078 |
| AT688_RS02090 | WP_005897292.1 | 50S ribosomal protein L14 | 2.65 | <0.01 | *rplN* FNP_1061 |
| AT688_RS02120 | WP_005897280.1 | 50S ribosomal protein L18 | 2.33 | <0.01 | *rplR* FNP_1055 |
| AT688_RS02135 | WP_005897276.1 | 50S ribosomal protein L15 | 2.39 | <0.01 | *rplO* FNP_1052 |
| AT688_RS10615 | WP_005894529.1 | 30S ribosomal protein S12 | 2.47 | <0.01 | *rpsL* FNP_2156 |
| **Transmembrane transport** | | | | | |
| AT688_RS00165 | WP_005895065.1 | type II/IV secretion system protein | -2.15 | 0.01 | FNP_2399 |
| AT688_RS00170 | WP_005895062.1 | type II secretion system F family protein | -3.43 | <0.01 | FNP_2398 |
| **Unkonwn function** | | | | | |
| AT688_RS06345 | WP_005896970.1 | inositol phosphorylceramide synthase | 6.72 | <0.01 | FNP_0885 |
| AT688_RS11315 | WP_005895403.1 | DUF1353 domain-containing protein | 6.39 | <0.05 | FNP_0107 |
| AT688_RS06515 | WP_005897067.1 | hypothetical protein | 6.17 | <0.01 | FNP_0921 |
| AT688_RS09595 | WP_005898661.1 | YARHG domain-containing protein | 5.56 | <0.01 | FNP_1989 |
| AT688_RS03335 | WP_005895754.1 | hypothetical protein | 4.61 | 0.02 | FNP_0267 |
| AT688_RS11820 | WP_011950376.1 | hypothetical protein | 5.29 | <0.01 | FNP_pFN3g02 |

**Table S6.** ***F. nucleatum*** **subsp.** ***polymorphum*** **DEGs enriched in key GO biological processes.**

| **Locus tag** | **Protein ID** | **Gene description** | **Fold change** | ***P* adj** | **Gene symbol/ old locus tag** |
|---|---|---|---|---|---|
| **Fourteen DEGs involved in five interrelated biological processes** | | | | | |
| AT688_RS00740 | WP_005894768.1 | 30S ribosomal protein S15 | 2.12 | <0.01 | *rpsO* FNP_2280 |
| AT688_RS02045 | WP_005897305.1 | 50S ribosomal protein L4 | 2.03 | <0.01 | *rplD* FNP_1070 |
| AT688_RS02050 | WP_005892379.1 | 50S ribosomal protein L23 | 2.03 | <0.01 | *rplW* FNP_1069 |
| AT688_RS02055 | WP_005897303.1 | 50S ribosomal protein L2 | 2.35 | <0.01 | *rplB* FNP_1068 |
| AT688_RS02075 | WP_005897296.1 | 50S ribosomal protein L16 | 2.27 | <0.01 | *rplP* FNP_1064 |
| AT688_RS02100 | WP_005897289.1 | 50S ribosomal protein L5 | 2.26 | <0.01 | *rplE* FNP_1059 |
| AT688_RS02125 | WP_005897278.1 | 30S ribosomal protein S5 | 2.41 | <0.01 | *rpsE* FNP_1054 |
| AT688_RS02130 | WP_005892817.1 | 50S ribosomal protein L30 | 2.33 | <0.01 | *rpmD* FNP_1053 |
| AT688_RS03600 | WP_005895834.1 | 16S rRNA processing protein RimM | -5.26 | <0.01 | *rimM* FNP_0319 |
| AT688_RS09940 | WP_005898763.1 | 30S ribosomal protein S16 | 2.61 | <0.01 | *rpsP* FNP_2060 |
| AT688_RS03845 | WP_005895940.1 | 50S ribosomal protein L20 | 2.61 | <0.01 | *rplT* FNP_0368 |
| AT688_RS05305 | WP_005896534.1 | 50S ribosomal protein L27 | 2.48 | <0.01 | *rpmA* FNP_0673 |
| AT688_RS09430 | WP_005898626.1 | 50S ribosomal protein L19 | 4.89 | <0.01 | *rplS* FNP_1955 |
| AT688_RS09690 | WP_005898701.1 | 50S ribosomal protein L32 | 2.69 | <0.01 | *rpmF* FNP_2008 |

**Table S7. *F. nucleatum* subsp. *polymorphum* DEGs enriched in key KEGG pathways.**

| Locus tag | Protein ID | Gene description | Fold change | *P* adj | Gene symbol/ old locus tag |
|---|---|---|---|---|---|
| **Translation** | | | | | |
| AT688_RS00740 | WP_005894768.1 | 30S ribosomal protein S15 | 2.12 | <0.01 | *rpsO* FNP_2280 |
| AT688_RS02005 | WP_005897324.1 | 30S ribosomal protein S18 | 2.71 | <0.01 | *rpsR* FNP_1078 |
| AT688_RS02045 | WP_005897305.1 | 50S ribosomal protein L4 | 2.03 | <0.01 | *rplD* FNP_1070 |
| AT688_RS02050 | WP_005892379.1 | 50S ribosomal protein L23 | 2.03 | <0.01 | *rplW* FNP_1069 |
| AT688_RS02055 | WP_005897303.1 | 50S ribosomal protein L2 | 2.35 | <0.01 | *rplB* FNP_1068 |
| AT688_RS02075 | WP_005897296.1 | 50S ribosomal protein L16 | 2.27 | <0.01 | *rplP* FNP_1064 |
| AT688_RS02090 | WP_005897292.1 | 50S ribosomal protein L14 | 2.65 | <0.01 | *rplN* FNP_1061 |
| AT688_RS02100 | WP_005897289.1 | 50S ribosomal protein L5 | 2.26 | <0.01 | *rplE* FNP_1059 |
| AT688_RS02120 | WP_005897280.1 | 50S ribosomal protein L18 | 2.33 | <0.01 | *rplR* FNP_1055 |
| AT688_RS02125 | WP_005897278.1 | 30S ribosomal protein S5 | 2.41 | <0.01 | *rpsE* FNP_1054 |
| AT688_RS02130 | WP_005892817.1 | 50S ribosomal protein L30 | 2.33 | <0.01 | *rpmD* FNP_1053 |
| AT688_RS02135 | WP_005897276.1 | 50S ribosomal protein L15 | 2.39 | <0.01 | *rplO* FNP_1052 |
| AT688_RS03845 | WP_005895940.1 | 50S ribosomal protein L20 | 2.61 | <0.01 | *rplT* FNP_0368 |
| AT688_RS05305 | WP_005896534.1 | 50S ribosomal protein L27 | 2.48 | <0.01 | *rpmA* FNP_0673 |
| AT688_RS09430 | WP_005898626.1 | 50S ribosomal protein L19 | 4.89 | <0.01 | *rplS* FNP_1955 |
| AT688_RS09690 | WP_005898701.1 | 50S ribosomal protein L32 | 2.69 | <0.01 | *rpmF* FNP_2008 |
| AT688_RS09940 | WP_005898763.1 | 30S ribosomal protein S16 | 2.61 | <0.01 | *rpsP* FNP_2060 |
| AT688_RS10615 | WP_005894529.1 | 30S ribosomal protein S12 | 2.47 | <0.01 | *rpsL* FNP_2156 |
| **Protein export** | | | | | |
| AT688_RS02140 | WP_005897274.1 | preprotein translocase subunit SecY | 2.56 | <0.01 | *secY* FNP_1051 |
| AT688_RS04215 | WP_005896104.1 | preprotein translocase subunit YajC | 3.45 | <0.01 | *yajC* FNP_0448 |
| AT688_RS08695 | WP_005898423.1 | preprotein translocase subunit SecG | 2.20 | <0.01 | *secG* FNP_1802 |
| AT688_RS11260 | WP_005895436.1 | lipoprotein signal peptidase | -3.55 | <0.01 | *lspA* FNP_0120 |

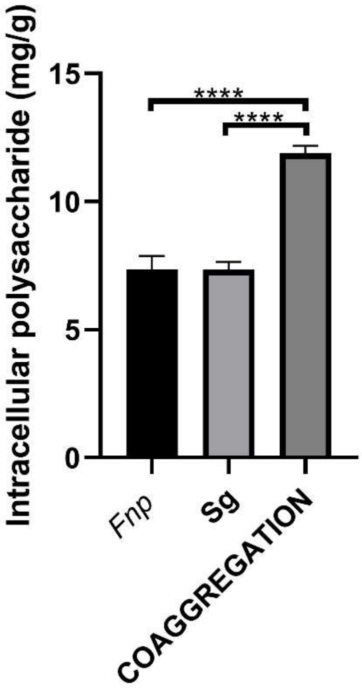


**Figure S1. Quantification of intracellular polysaccharide content.**

Mono-cultures and coaggregates were cultured with BHI broth supplemented with 1% sucrose for 2 h, respcetively. Glycogen content was determined using Glycogen Content Assay Kit (Solarbio, Beijing, China). Data represent the mean ± SD of the results of three independent assays. The asterisks indicate significant difference between groups ($p$ < 0.05).
